# Solid-state ^13^C-NMR spectroscopic determination of side-chain mobilities in zirconium-based metal–organic frameworks

**DOI:** 10.5194/mr-5-1-2024

**Published:** 2024-01-05

**Authors:** Günter Hempel, Ricardo Kurz, Silvia Paasch, Kay Saalwächter, Eike Brunner

**Affiliations:** 1 Martin-Luther-Universität Halle-Wittenberg, Institut für Physik – NMR, Betty-Heimann-Str. 7, 06120 Halle, Germany; 2 Technische Universität Dresden, Fakultät für Chemie und Lebensmittelchemie, Bioanalytische Chemie, 01062 Dresden, Germany

## Abstract

Porous interpenetrated zirconium–organic frameworks (PIZOFs) are a class of Zr-based metal–organic frameworks (MOFs) which are composed of long, rod-like dicarboxylate linkers and ${\mathrm{Zr}}_{6}O_{4}(\mathrm{OH}{)}_{4}(O_{2}C{)}_{12}$ nodes. Long oligoethylene glycol or aliphatic side chains are covalently attached to the linker molecules in the cases of PIZOF-10 and PIZOF-11, respectively. These side chains are supposedly highly mobile, thus mimicking a solvent environment. It is anticipated that such MOFs could be used as a solid catalyst – the MOF – with pore systems showing properties similar to a liquid reaction medium. To quantify the side-chain mobility, here we have applied different 1D and 2D NMR solid-state spectroscopic techniques like cross-polarization (CP) and dipolar-coupling chemical-shift correlation (DIPSHIFT) studies. The rather high ${}^{1}H$ - ${}^{13}C$ CP efficiency observed for the ${\mathrm{CH}}_{2}$ groups of the side chains indicates that the long side chains are unexpectedly immobile or at least that their motions are strongly anisotropic. More detailed information about the mobility of the side chains was then obtained from DIPSHIFT experiments. Analytical expressions for elaborate data analysis are derived. These expressions are used to correlate order parameters and to slow motional rates with signals in indirect spectral dimensions, thus enabling the quantification of order parameters for the ${\mathrm{CH}}_{2}$ groups. The ends of the chains are rather mobile, whereas the carbon atoms close to the linker are more spatially restricted in mobility.

## 1 Introduction

Metal–organic frameworks (MOFs) are crystalline porous materials [Bibr bib1.bibx26] composed of organic and inorganic building units forming networks with micropores and/or mesopores. Due to promising properties such as an extraordinarily high specific surface area and gas storage capacity, MOFs are assumed to find numerous applications, e.g. in gas storage and separation, drug delivery, sensing, and wastewater treatment [Bibr bib1.bibx21]. Favourable MOFs for catalysis [Bibr bib1.bibx45] should be stable at elevated temperatures and in the presence of moisture. Most members of the PIZOF (porous interpenetrated zirconium–organic framework) family [Bibr bib1.bibx52] are very stable and insensitive to moisture, in contrast to other MOFs containing e.g. Zn instead of Zr (Kaye et al., 2007; Feng et al., 2010), which makes this type of MOF particularly interesting for catalytic applications. PIZOFs contain long dicarboxylates as linkers (see Fig. [Fig Ch1.F1]) and ${\mathrm{Zr}}_{6}O_{4}(\mathrm{OH}{)}_{4}(O_{2}C{)}_{12}$ as nodes. The surface properties of the internal pore system, including its polarity, can be tuned in PIZOFs by adding appropriate side chains to the linkers. Examples are PIZOF-10 and PIZOF-11 (see Fig. [Fig Ch1.F1]). Both exhibit high thermal stability and resistance against atmospheric moisture like other members of the PIZOF family [Bibr bib1.bibx34]. The aliphatic side chains make the internal surface of PIZOF-11 hydrophobic, whereas PIZOF-10 with its oligoethylene glycol side chains is rather hydrophilic.

**Figure 1 Ch1.F1:**
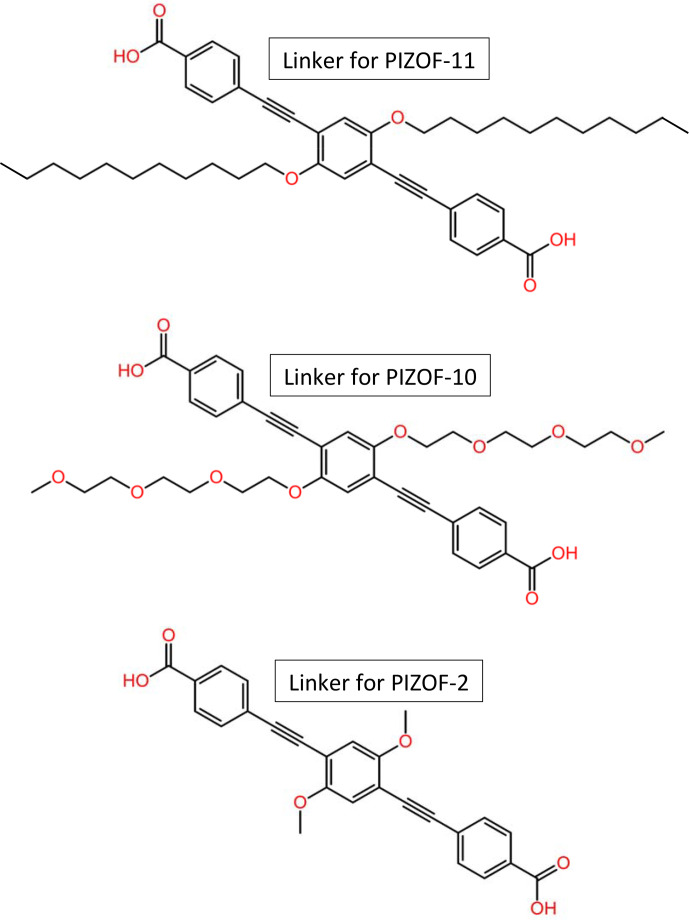
Structural formulae of the linkers for the herein studied PIZOFs: PIZOF-2, PIZOF-10, and PIZOF-11.

The long oligoethylene glycol or aliphatic side chains in PIZOF-10 and PIZOF-11 are supposed to be highly flexible, thus providing a liquid-like environment in the remaining pores. In order to test this hypothesis, solid-state NMR techniques were applied to evaluate the side-chain mobility. Solid-state NMR spectroscopy is a powerful tool for determining structural parameters and detecting mobile structural elements in biological systems as well as various materials [Bibr bib1.bibx50]. The framework structure of MOFs can be studied in general by ${}^{1}H$ , ${}^{13}C$ , or ${}^{17}O$ magic-angle spinning (MAS) NMR spectroscopy [Bibr bib1.bibx36]. Furthermore, nuclei such as ${}^{27}\mathrm{Al}$ [Bibr bib1.bibx23], ${}^{71}\mathrm{Ga}$ [Bibr bib1.bibx58], ${}^{45}\mathrm{Sc}$ [Bibr bib1.bibx42], and others [Bibr bib1.bibx16] can be used in order to detect the environment of the central metal atom. NMR spectroscopy also allows the study of host or guest interactions with adsorbed species [Bibr bib1.bibx61].

It is well known that the efficiency of the cross-polarization (CP) experiment [Bibr bib1.bibx47] is strongly influenced by the presence of thermal motions [Bibr bib1.bibx54]. This was exploited previously in order to qualitatively characterize the mobility of aliphatic chains, e.g. of n-alkylsilanes bonded to silica surfaces [Bibr bib1.bibx56], surfactants in mesoporous MCM-41 materials [Bibr bib1.bibx55], and alkanes grafted to silica for chromatography [Bibr bib1.bibx48].

The motion of the side chains is expected to be anisotropic because of its fixation at the middle ring of the linker backbone. The degree of anisotropy (expressed e.g. by the order parameter S ) resolved for individual atomic positions might be an important characteristic of the chain motion. More detailed information can be obtained by some 2D NMR methods. In the work presented here, the DIPSHIFT (dipolar-coupling chemical-shift correlation) technique was applied in order to site-specifically determine order parameters. A pulse scheme of this sequence is shown in the Supplement. It was developed as a separated local-field experiment under MAS [Bibr bib1.bibx43]. Applications made use of the possibility of determining the residual dipolar coupling site-specifically [Bibr bib1.bibx9], and a recent paper compared the results with those of rotational echo double resonance REDOR,. The DIPSHIFT experiment enables correlation of isotropic chemical shifts, i.e. atomic positions, in direct dimension ( $t_{2}$ ) with signals in the indirect dimension ( $t_{1}$ ) corresponding to an FID which solely evolves under MAS-modulated ${}^{1}H$ – ${}^{13}C$ dipolar couplings between subsequent rotational echoes. The latter is true under the condition that protons coupled to the considered nucleus do not undergo interactions with each other and provided that Fourier transform is performed in the direct dimension only. The depth of the minimum between consecutive echoes is a measure of the (residual) dipolar coupling, which itself contains information about the order parameter. The decrease in the second echo with respect to the first one represents the influence of intermediate motions (intermediate-motional $T_{2}$ effect).

To determine the mobility site-selectively, ${}^{13}C$ signal assignment was necessary. This was achieved by the application of techniques such as solid-state attached proton test (APT, [Bibr bib1.bibx31]), ${}^{1}H$ - ${}^{13}C$ heteronuclear correlation (HETCOR), and ${}^{1}H$ - ${}^{13}C$ heteronuclear multiple-quantum coherence HMQC,.

The analysis of DIPSHIFT data was in the past usually performed by comparison with numerically calculated data under parameter variation. In this paper, analytical expressions were derived and used. This allowed us to estimate the parameter set for best data fitting in a more efficient way. Intermediate motions were included in the analytical expressions by application of the Anderson–Weiss procedure [Bibr bib1.bibx2]. Specifically, we focus on the conditions under which the signal damping arising from intermediate motions can be described by an exponential function. The theoretical background of this procedure is described in the next section.

## 2 Equations and models for DIPSHIFT data evaluation

### 2.1 Derivation

MAS experiments on samples with anisotropic spin interactions result in spectra with spinning sidebands (SSBs) and in the FIDs containing echo trains. Provided that the anisotropic interaction is described by a second-order tensor, the SSB intensities can be efficiently calculated by polynomial expressions following [Bibr bib1.bibx17]. Equation (21) in that paper gives the general equation for the intensity $S_{m}(D/\omega_{r},\eta )$ of the m th-order sideband; the parameters in the argument list are the anisotropy D of the tensor, the angular spinning frequency $\omega_{r}$ , and the asymmetry parameter η of the tensor. A closed-form evaluation of this equation is hardly possible; equations for SSBs which are ready for use must be obtained by symbolic-language software. Examples of low-order SSBs are given in the mentioned paper.

The FID can be calculated as Fourier synthesis if the SSB intensities are known.

1 $$F(t)=\sum_{m=-\infty }^{\infty }S_{m}\left(\frac{D}{\omega_{r}},\eta \right)e^{im\omega_{r}t}$$

Applying this formalism to spin systems where chemical-shift anisotropy is the dominating interaction is straightforward. In the case of dipolar interaction, however, some special aspects have to be considered.

- Fast anisotropic thermal motion: the tensor is subjected to a partial averaging process over all possible states which occur during this motion. Then, the anisotropy and asymmetry parameters of this averaged tensor have to be inserted into the SSB polynomials. In this paper, this case has to be considered for modelling two-site jumps.
- Dipolar interaction with more than one spin: tensors of the individual couplings have to be added up. If, however, the neighbouring spins interact among themselves, it is possible that the total interaction is represented by a tensor of order >2 . In the latter case the polynomial formulae mentioned above would not be applicable.
- In the case of multi-spin interactions, different combinations of spin orientations have to be taken into account; they can lead to different effective tensors. Each of these tensors corresponds to a particular set of SSB $\left\{S_{m}^{k}\right\}$ , where k indicates the respective combinations of spin orientations. The SSB intensity which appears in the experiment is the average of the SSB intensities belonging to all the combinations: $S_{m}=\sum_{k}S_{m}^{k}$ . This procedure is applied below for the calculation of FIDs of both the ${\mathrm{CH}}_{2}$ and ${\mathrm{CH}}_{3}$ groups.

### 2.2 Transformation to a cosine series

In this paper we consider dipolar interactions between two nuclei with spin 1/2 . (“ 1/2 ” should be understood as “one-half”.) The observed spin can be oriented parallel or antiparallel to $B_{0}$ with approximately equal probabilities at not too low temperatures. A change in orientation of the observed spin inverts the anisotropy of the dipolar tensor assuming the configuration of the coupled spins remains unchanged. We thus always have pairs of tensors with inverted anisotropy ( D and -D ) but equal asymmetry parameters. Applying the relation see,

2 $$S_{m}\left(\frac{D}{\omega_{r}},\eta \right)=S_{-m}\left(-\frac{D}{\omega_{r}},\eta \right),$$

the exponential series of Eq. ([Disp-formula Ch1.E1]) can always be transformed to a cosine series,

3 $$F(t)=\sum_{m=0}^{\infty }C_{m}\mathrm{cos}m\omega_{r}t,$$

with the following definitions:

4 $$\begin{array}{rl} C_{0}:=S_{0},\\ C_{m}\left(\frac{D}{\omega_{r}},\eta \right):= & S_{m}\left(\frac{D}{\omega_{r}},\eta \right)+S_{-m}\left(\frac{D}{\omega_{r}},\eta \right)\\ = & S_{m}\left(\frac{D}{\omega_{r}},\eta \right)+S_{m}\left(-\frac{D}{\omega_{r}},\eta \right). \end{array}$$

Therefore, the $C_{m}$ contain only even powers of $D/\omega_{r}$ .

In the following, the polynomial equations are established for four situations which may occur in the PIZOF samples.

### 2.3 Model 1: ensemble of isolated spin pairs

The coefficients are denoted by $C_{m}^{\text{IS}}$ and can be taken immediately from the generic equation by using the dipolar-coupling constant $D_{0}$ as the anisotropy and zero asymmetry parameter. As an example, up to 12th order, we have

5 $$\begin{array}{rl} C_{0}^{\text{IS}}=1 & -\frac{3}{20}{\left(\frac{D_{0}}{\omega_{r}}\right)}^{2}+\frac{227}{181\;440}{\left(\frac{D_{0}}{\omega_{r}}\right)}^{4}\\ & -\frac{1103}{2\;306\;304}{\left(\frac{D_{0}}{\omega_{r}}\right)}^{6}+\frac{22\;859}{1\;792\;327\;680}{\left(\frac{D_{0}}{\omega_{r}}\right)}^{8}\\ & -\frac{3\;308\;407}{14\;302\;774\;886\;400}{\left(\frac{D_{0}}{\omega_{r}}\right)}^{10}\\ & +\frac{108\;665\;671}{35\;886\;962\;442\;240\;000}{\left(\frac{D_{0}}{\omega_{r}}\right)}^{12}. \end{array}$$

The polynomial equations for m=1 to 3 are given in Sect. [Sec Ch1.S6.SS1].

### 2.4 Model 2: spin pairs with fast two-site jumps

“Fast” means that the motional average is already complete at times which are relevant for the experiment. For the DIPSHIFT experiments, this is the time between the end of excitation and the beginning of data acquisition. Thus, the averaging must already be complete at the first time step in the indirect dimension, which is 1 order of magnitude smaller than the rotation period, i.e. about 20 µs . In the case of cross-polarization, this relevant time is of the order of the reciprocal of the dipolar-coupling constant, i.e. also about $10^{-5}$ s. The coefficients for model 2 are denoted by $C_{m}^{\text{IS-j}}$ . The characteristic tensor is the average 〈D〉 of the tensors of both sites $D_{1}$ and $D_{2}$ : see Fig. [Fig Ch1.F2]. We suppose that the sites are occupied with equal probability. The angle between the dipolar axes of both sites is 2α . In a frame where the z axis is along the bisector, the y axis is perpendicular to the plane spanned by the IS connection vectors of both sites, the x axis is in this plane perpendicular to the bisector, and both tensors are represented by the matrices $D_{1}$ and $D_{2}$ as

6 $$D_{1;2}=D_{0}\left(\begin{array}{ccc} \frac{1}{2}-P_{2}\left(\mathrm{cos}\alpha \right) & 0 & \pm \frac{3}{4}\mathrm{sin}2\alpha \\ 0 & -\frac{1}{2} & 0\\ \pm \frac{3}{4}\mathrm{sin}2\alpha & 0 & P_{2}\left(\mathrm{cos}\alpha \right) \end{array}\right).$$

The average of both matrices is diagonal in this bisector frame:

7 $$\langle D\rangle =D_{0}\left(\begin{array}{ccc} \frac{1}{2}-P_{2}\left(\mathrm{cos}\alpha \right) & 0 & 0\\ 0 & -\frac{1}{2} & 0\\ 0 & 0 & P_{2}\left(\mathrm{cos}\alpha \right) \end{array}\right).$$

In particular, in this work, 180° ring flips are of interest. This means that the flip angle for the CH bonds is 2α=120° :

8 $$\langle D\rangle_{120{}^{\circ}}=D_{0}\left(\begin{array}{ccc} \frac{5}{8} & 0 & 0\\ 0 & -\frac{1}{2} & 0\\ 0 & 0 & -\frac{1}{8} \end{array}\right).$$

In the latter case, the anisotropy is $\frac{5}{8}D_{0}$ and the magnitude of the asymmetry parameter is $\eta =\frac{3}{5}$ . The cos-Fourier coefficients are

9 $$C_{m}^{\text{IS-j}}=C_{m}\left(\frac{5D_{0}}{8\omega_{r}},\frac{3}{5}\right).$$

The explicit polynomial equations can be found in Sect. [Sec Ch1.S6.SS1].

**Figure 2 Ch1.F2:**
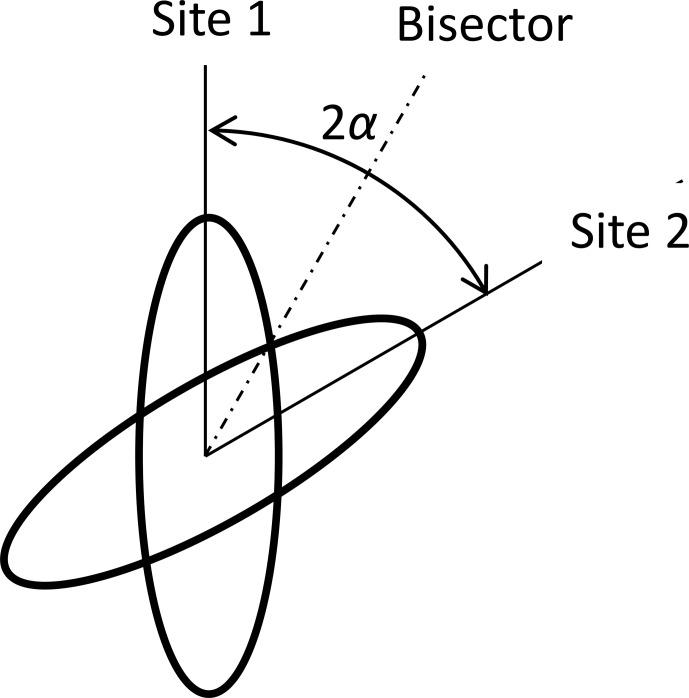
Two-site jumps between positions differing by an angle of 2α .

### 2.5 Model 3: IS_2_ spin system

Here it is assumed that the observed spin I interacts with two neighbouring spins S . The coupling constant for each single coupling is equal to $D_{0}$ , i.e. the distances between I , and both S are assumed to be equal. There are four combinations of the S spin orientations. The two combinations where the two $S_{z}$ are parallel generate the tensor $D_{\Sigma }$ , and the two with antiparallel $S_{z}$ generate the tensor $D_{\Delta }$ . In the bisector frame (see Fig. [Fig Ch1.F3]), the tensor matrices are the same as in Eq. ([Disp-formula Ch1.E6]). The matrices of the sum and difference tensors for tetrahedral symmetry are

10 $$\begin{array}{rl} & D_{\Sigma }=D_{0}\left(\begin{array}{ccc} 1 & 0 & 0\\ 0 & -1 & 0\\ 0 & 0 & 0 \end{array}\right),\\ & D_{\Delta }=D_{0}\left(\begin{array}{ccc} 0 & 0 & \sqrt{2}\\ 0 & 0 & 0\\ \sqrt{2} & 0 & 0 \end{array}\right), \end{array}$$

respectively. From $D_{\Sigma }$ we can read off directly the anisotropy as $D_{0}$ and the asymmetry parameter η=1 . $D_{\Delta }$ has an eigenvalue vector $\left(0,D_{0}\sqrt{2},-D_{0}\sqrt{2}\right)$ which gives anisotropy $D_{0}\sqrt{2}$ and η=1 . Hence the cosine coefficients for a tetrahedral IS_2_ spin system are

11 $$C_{m}^{\text{IS2}}=\frac{1}{2}\left[C_{m}\left(\frac{D_{0}}{\omega_{r}},1\right)+C_{m}\left(\frac{D_{0}\sqrt{2}}{\omega_{r}},1\right)\right].$$

The polynomial equations can again be found in Sect. [Sec Ch1.S6.SS1].

**Figure 3 Ch1.F3:**
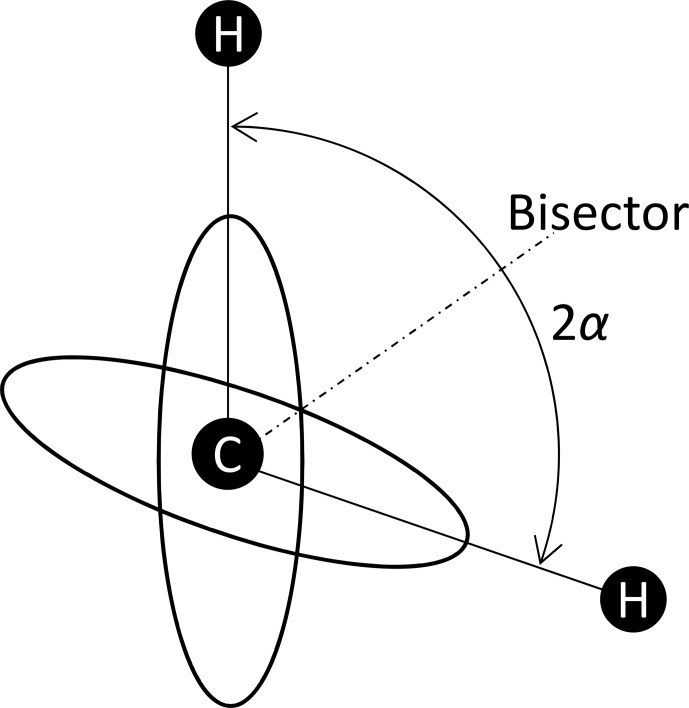
${}^{1}H$ positions within a ${\mathrm{CH}}_{2}$ group. The angle between the C–H connection vectors is 2α . Supposing tetrahedral symmetry, $\alpha =\mathrm{arccos}(1/\sqrt{3})$ .

### 2.6 Model 4: rapidly rotating methyl groups

Here we consider the special case of an IS_3_ spin system. The observed spin I resides in the middle of a tetrahedron, the three IS bonds have equal lengths and point to three corners of this tetrahedron, and the rotation axis points to the remaining corner: see Fig. [Fig Ch1.F4]. The tensor of a single CH coupling in a frame, the z axis of which is along the rotation axis, has the matrix

12 $$D_{1}=D_{0}\left(\begin{array}{ccc} \frac{1}{6}+\frac{2}{3}\mathrm{cos}2\varphi & \frac{2}{3}\mathrm{sin}2\varphi & \frac{\sqrt{2}}{3}\mathrm{cos}\varphi \\ \frac{2}{3}\mathrm{sin}2\varphi & \frac{1}{6}-\frac{2}{3}\mathrm{cos}2\varphi & \frac{2}{3}\mathrm{sin}2\varphi \\ \frac{\sqrt{2}}{3}\mathrm{cos}\varphi & \frac{2}{3}\mathrm{sin}2\varphi & -\frac{1}{3} \end{array}\right),$$

where φ is the instantaneous rotation angle around the z axis. Averaging over this angle, which is equivalent to fast-limit time averaging corresponding to a methyl group at not too low temperatures, gives for all three couplings a tensor matrix which is diagonal in this frame:

13 $$\langle D\rangle_{\varphi }=D_{0}\left(\begin{array}{ccc} 1/6 & 0 & 0\\ 0 & 1/6 & 0\\ 0 & 0 & -1/3 \end{array}\right).$$

There are eight spin orientation permutations of the three S spins: see Table [Table Ch1.T1].

**Figure 4 Ch1.F4:**
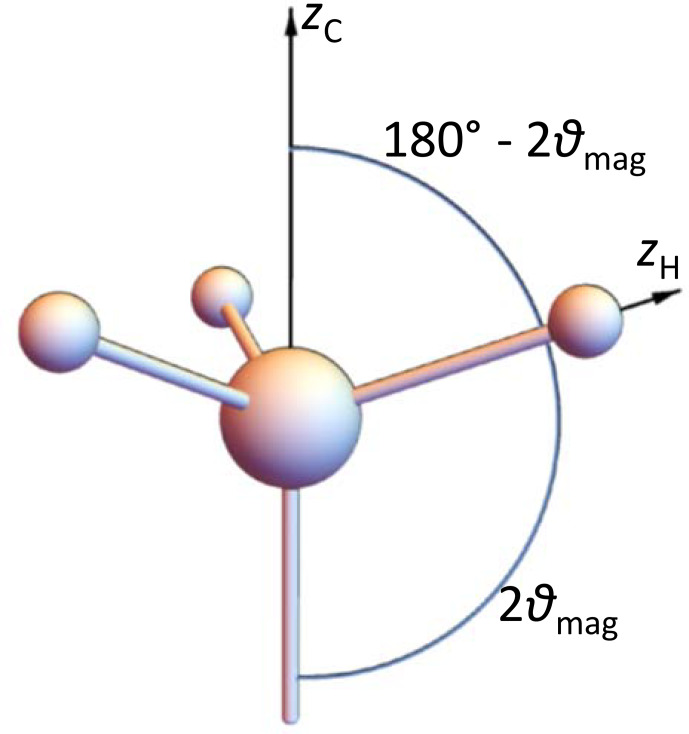
Geometry of a ${\mathrm{CH}}_{3}$ group.

**Table 1 Ch1.T1:** All possibilities of combining the orientations of the three proton spins in a ${\mathrm{CH}}_{3}$ group. The anisotropy and matrix of the sum tensor are displayed in the neighbouring columns.

Spins	Total tensor	Sum matrix	Anisotropy	η
↑↑↑	${\text{D}}_{1}+{\text{D}}_{2}+{\text{D}}_{3}$	$3\langle D\rangle_{\varphi }$	$-D_{0}$	0
↓↑↑	$-{\text{D}}_{1}+{\text{D}}_{2}+{\text{D}}_{3}$	$\langle D\rangle_{\varphi }$	$-D_{0}/3$	0
↑↓↑	${\text{D}}_{1}-{\text{D}}_{2}+{\text{D}}_{3}$	$\langle D\rangle_{\varphi }$	$-D_{0}/3$	0
↑↑↓	${\text{D}}_{1}+{\text{D}}_{2}-{\text{D}}_{3}$	$\langle D\rangle_{\varphi }$	$-D_{0}/3$	0
↑↓↓	${\text{D}}_{1}-{\text{D}}_{2}-{\text{D}}_{3}$	$-\langle D\rangle_{\varphi }$	$D_{0}/3$	0
↓↑↓	$-{\text{D}}_{1}+{\text{D}}_{2}-{\text{D}}_{3}$	$-\langle D\rangle_{\varphi }$	$D_{0}/3$	0
↓↓↑	$-{\text{D}}_{1}-{\text{D}}_{2}+{\text{D}}_{3}$	$-\langle D\rangle_{\varphi }$	$D_{0}/3$	0
↓↓↓	$-{\text{D}}_{1}-{\text{D}}_{2}-{\text{D}}_{3}$	$-3\langle D\rangle_{\varphi }$	$D_{0}$	0

Considering that the anisotropy enters the $C_{m}$ equations in even powers only, the cosine coefficients are

14 $$C_{m}^{\text{CH3}}=\frac{1}{4}C_{m}\left(\frac{D_{0}}{\omega_{r}},0\right)+\frac{3}{4}C_{m}\left(\frac{D_{0}}{3\omega_{r}},0\right).$$

The explicit polynomial equations can again be found in Sect. [Sec Ch1.S6.SS1].

### 2.7 Influence of remote protons

Because of the $r^{-3}$ dependence of the dipolar coupling, the dipolar environment of protonated carbons is essentially determined by the directly bound proton(s) located at a distance of about 1.1 Å. The situation is less unique for non-protonated carbons, where several remote protons with similar distances to the regarded carbon are often relevant. Then, many details of the molecular geometry have to be considered to establish a model function. On the other hand, a restriction to allegedly dominant protons can be a reason for incorrect data analysis. As an attempt, the cosine coefficients $C_{m}$ were calculated for the acetylenic carbons considering the closest protons of each neighbouring ring. Comparing this model function with experimental data, however, showed that the former does not reflect the real situation; the residual couplings obtained by the analysis are still larger than the full coupling expected from the model. Consequently, we restricted the DIPSHIFT evaluations to protonated carbons.

### 2.8 Fast anisotropic motion

As mentioned above, fast-limit averaging means that motional averaging is complete at times which are relevant for the experiment. The effect of fast-motional averaging can be described by tensor matrices with elements changed compared to the rigid case, hence also the anisotropy and asymmetry parameter change. Two general cases can be distinguished.

- All three eigenvalues are decreased by a common factor which is called order parameter S . Then, the anisotropy is also reduced by the same factor; the asymmetry parameter stays constant. Variable $D_{0}$ in the functions from above is then replaced by the residual dipolar coupling $D_{\text{res}}=S\cdot D_{0}$ . Relevant examples are uniaxial rotation or rotational jumps between three or more equally distributed and populated sites.
- The tensor eigenvalues change during the averaging process in a different manner. Then the introduction of a single-order parameter will not provide an adequate description of the situation, anisotropy and asymmetry parameter change non-trivially, and a new function has to be used. As an example, consider the above case of two-site jumps. The function $C_{m}^{\text{IS}}$ had to be replaced by $C_{m}^{\text{IS-j}}$ . This means that the asymmetry is reduced, but at the same time the asymmetry parameter increases from 0 (case of $C_{m}^{\text{IS}}$ ) to 3/5 (case of $C_{m}^{\text{IS-j}}$ ). Thus, the tensor principal values of one case can no longer be represented as common multiples of the principal values of the other.

### 2.9 Intermediate motions

Intermediate motions, i.e. motions with a rate of range of the static-limit dipolar-coupling constant and/or the spinning frequency, interfere with the refocusing effect of MAS. This manifests itself in a damping of the rotational echoes or even in their complete disappearance. A rather weak echo damping is observed in our data sets. To analyze these, we need an analytical expression for this case, for which we took the heuristic approach of multiplying the rigid-lattice model functions derived above by an exponential damping function. The relevant questions are those conditions under which this can be an appropriate approximation and whether the fitted decay constant contains information on the motional timescale.

An approximate analytical expression for the MAS FID $F_{\text{AW}}(t)$ during slow or intermediate motion was derived by [Bibr bib1.bibx19] using the Anderson–Weiss (AW) formalism [Bibr bib1.bibx2]:

15 $$F_{\text{AW}}(t)=\mathrm{exp}\left\{-\int_{0}^{t}\left(t-\tau \right)K(\tau )d\tau \right\}.$$

K(τ) is the orientation autocorrelation function of the motion under consideration. In the case of MAS, the expression derived by [Bibr bib1.bibx7]

16 $$K(\tau )=e^{-\tau /\tau_{c}}\left(\frac{2}{3}\mathrm{cos}\omega_{r}\tau +\frac{1}{3}\mathrm{sin}\omega_{r}\tau \right),$$

containing an assumed exponential decay describing the random motion with correlation time $\tau_{c}$ and multiplied by the coherent (oscillatory) correlation function of the MAS rotation, was inserted into Eq. ([Disp-formula Ch1.E15]). This gave the following.

17 $$\begin{array}{rl} F_{\text{AW}}(t) & =\mathrm{exp}\left\{-\frac{1}{3}M_{2}t\left(\frac{2\tau_{c}}{1+{\left(\omega_{r}\tau_{c}\right)}^{2}}+\frac{\tau_{c}}{1+4{\left(\omega_{r}\tau_{c}\right)}^{2}}\right)\right.\\ & +\frac{1}{3}M_{2}\tau_{c}^{2}\left[2\frac{1-{\left(\omega_{r}\tau_{c}\right)}^{2}}{{\left(1+{\left(\omega_{r}\tau_{c}\right)}^{2}\right)}^{2}}\left(1-e^{-t/\tau_{c}}\mathrm{cos}\omega_{r}t\right)\right.\\ & +\left.\frac{1-4{\left(\omega_{r}\tau_{c}\right)}^{2}}{{\left(1+4{\left(\omega_{r}\tau_{c}\right)}^{2}\right)}^{2}}\left(1-e^{-t/\tau_{c}}\mathrm{cos}2\omega_{r}t\right)\right]\\ & +\left.\frac{4}{3}M_{2}\tau_{c}^{2}e^{-t/\tau_{c}}\left[\frac{\mathrm{sin}\omega_{r}t}{{\left(1+{\left(\omega_{r}\tau_{c}\right)}^{2}\right)}^{2}}+\frac{\mathrm{sin}2\omega_{r}t}{{\left(1+4{\left(\omega_{r}\tau_{c}\right)}^{2}\right)}^{2}}\right]\right\} \end{array}$$

This equation describes a dipolar FID, which is an echo train for large $\tau_{c}$ or a monotonous decay for fast motion, i.e. short $\tau_{c}$ . The echo train structure is already widely lost in the vicinity of $\omega_{r}\tau_{c}=1$ . However, it is an approximate expression because the AW approach restricts the cumulant expansion of the distribution of the reduced phase $\varphi :=(1/t)\int_{0}^{t}\omega (t_{1})dt_{1}$ to the second-order term. Hence, all reduced-phase distributions are regarded as Gaussian distributions within the AW treatment because only for Gaussian distributions do all cumulants with order >2 disappear. This could be a good approximation for short times ( $t\ll M_{2}^{-1/2}$ ) [Bibr bib1.bibx9] as well as for long times ( $t\gg \tau_{c}$ ). The reason for this is that, (i) for short times, higher cumulants (order 2 n ) are still without influence because they are connected to $t^{2n}/(2n)!$ and, (ii) for long times, the reduced-phase distribution changes gradually toward a Gaussian distribution. The latter can be rationalized by means of the central limiting theorem, which states that the cumulants of order >2 decrease if a stochastic variable (here reduced phase) is the sum or integral of another stochastic variable (here frequency).

In between, we expect a “gap of validity”. Obviously, for sufficiently short correlation times ( $\tau_{c}\ll M_{2}^{-1/2}$ ) there is an overlap of both regions; here a gap of validity does not appear. For a rigid lattice, however, this gap ranges formally until infinity. For DIPSHIFT applications we have to consider that the data are usually plotted vs. $t_{1}/T_{r}=\omega_{r}t_{1}/(2\pi )$ in the interval [0,1] . To ensure that this interval does not reach essentially into the validity gap, $maxt_{1}=T_{r}$ should be smaller than $\sqrt{5}/D_{0}$ . In other words, $D_{0}/\omega_{r}<\approx 0.36$ . In fact, a numerical comparison of model function 1 (Eq. [Disp-formula Ch1.E5]) with Eq. ([Disp-formula Ch1.E17]) for $\tau_{c}\to \infty$ and $M_{2}\to D_{0}^{2}/5$ shows that deviations become relevant if this threshold is exceeded.

[Bibr bib1.bibx9] investigated the quality of the AW approximation for the DIPSHIFT experiment by comparison of Eq. ([Disp-formula Ch1.E17]) with numerically calculated data. Their results show that the deviations between Eq. ([Disp-formula Ch1.E17]) and experimental data are maximum in the intermediate region. [Bibr bib1.bibx8] did that in a similar manner for a related (re-coupled) experiment (rec-DIPSHIFT). This concerns situations where the echo structure of the FID is damped strongly.

Conversely, if we observe a weak damping of the echo train, we may conclude that the influence of intermediate motions is negligible (except for fast motions: see above). This means that, in this case, $\omega_{r}\tau_{c}\gg 1$ . With this condition, Eq. ([Disp-formula Ch1.E17]) can be simplified to

18 $$\begin{array}{rl} F_{\text{AW}}(t) & \approx F_{\text{rigid}}(t)\cdot \mathrm{exp}\left\{-\frac{t}{T_{\text{2MAS}}}\right\}\\ & =F_{\text{rigid}}(t)\cdot \mathrm{exp}\left\{-\frac{1}{f_{r}T_{\text{2MAS}}}\cdot \frac{t}{T_{r}}\right\}, \end{array}$$

where $F_{\text{rigid}}(t)$ denotes the FID without slow thermal motion and $f_{r}$ is the spinning frequency of the sample. The damping constant is defined as

19 $$\frac{1}{T_{\text{2MAS}}}:=\frac{3}{4}\frac{M_{2}}{\omega_{r}^{2}\tau_{c}}.$$

It is notable that the damping constant is proportional to $f_{r}^{-2}$ in a plot vs. t and is even proportional to $f_{r}^{-3}$ in the DIPSHIFT typical plot vs. $t/T_{r}$ . Numerical studies showed that the condition $\tau_{c}\ll T_{r}$ is tied to the intensity of the first rotational echo being at least half of the initial value.

The static part of Eq. ([Disp-formula Ch1.E17]) is obtained for $\tau_{c}\to \infty$ as

20 $$\begin{array}{rl} & F_{\text{rigid,AW}}=\\ & \mathrm{exp}\left\{-\frac{M_{2}}{3\omega_{r}^{2}}\left[2(1-\mathrm{cos}\omega_{r}t)+\frac{1}{4}(1-\mathrm{cos}2\omega_{r}t)\right]\right\} \end{array}.$$

We have confirmed numerically that any relevant deviations from the polynomial formulae occur if $D_{0}/\omega_{r}$ is comparable to or larger than 0.5. Consequently, $F_{\text{rigid}}(t)$ in Eq. ([Disp-formula Ch1.E18]) is represented by Eq. ([Disp-formula Ch1.E3]), where the $C_{m}$ are the corresponding coefficients derived for the four models shown above.

### 2.10 Second moment

The equations derived by the AW approach contain the second moment $M_{2}$ of the overall dipolar interactions.

21 $$M_{2}\equiv \langle \omega^{2}\rangle =\int \omega^{2}S(\omega )d\omega$$

S(ω) is the distribution of the frequency deviations from the centre of the resonance. In other words, it is the line-shape function. An ensemble of spins in the sample with equal atomic positions and equal spatial orientations (polar coordinates {θ,φ} of the z axis of the tensor main frame) contributes to the spectrum at the two positions:

22 $$\omega_{\text{aniso}}=\pm \frac{D}{2}\left[3{\mathrm{cos}}^{2}\theta -1+\eta {\mathrm{sin}}^{2}\theta \mathrm{cos}2\varphi \right].$$

The whole line-shape function is the sum over all individual lines which are assumed here to be δ functions:

23 $$S(\omega )=\int_{0}^{2\pi }d\varphi \int_{0}^{\pi }d\theta \mathrm{sin}\theta \delta \left(\omega -\omega_{\text{aniso}}(\theta ,\varphi )\right).$$

Inserting Eqs. ([Disp-formula Ch1.E23]) and ([Disp-formula Ch1.E22]) into Eq. ([Disp-formula Ch1.E21]) gives

24 $$M_{2}=\frac{D_{0}^{2}}{5}\left(1+\frac{\eta^{2}}{3}\right).$$

Now we can use the effective anisotropies and asymmetry parameters of the four models discussed above and obtain

25 $$\begin{array}{rl} & M_{2}^{\text{(CH)}}=\frac{D_{0}^{2}}{5},M_{2}^{\text{(CH-j)}}=\frac{7}{80}D_{0}^{2},M_{2}^{\text{(CH2)}}=\frac{2}{5}D_{0}^{2},\\ & M_{2}^{\text{(CH3)}}=\frac{1}{15}D_{0}^{2}. \end{array}$$

The last two expressions were obtained as averages between the corresponding expressions from both the sum and difference tensors mentioned above. They are equal to the expressions for the second moments of multi-spin dipolar interactions; in the case of ${\mathrm{CH}}_{3}$ , the fast rotation of this group was considered.

### 2.11 Parameter fit to experimental data

From the model functions and the exponential damping approximation, the following expressions can be obtained that are immediately applicable for the data fit in the four respective cases mentioned above ( $r:=\tau_{c}^{-1}$ ).

F1IS(Dres,ωr,r,t)=exp⁡-353πDres2rωr3tTr26×∑m=0mmaxCmISDresωr⋅cos⁡2πmtTrF2IS-j(Dres,ωr,r,t)=exp⁡-21803πDres2rωr3tTr27×∑m=0mmaxCmIS-jDresωr⋅cos⁡2πmtTrF3IS2(Dres,ωr,r,t)=exp⁡-653πDres2rωr3tTr28×∑m=0mmaxCmIS2Dresωr⋅cos⁡2πmtTrF4CH3(Dres,ωr,r,t)=exp⁡-153πDres2rωr3tTr29×∑m=0mmaxCmCH3Dresωr⋅cos⁡2πmtTr

For each sample, data were recorded at different temperatures and different spinning speeds $f_{n}$ . For each spinning speed, eight data points were recorded along the indirect dimension $t_{1}$ at times $0,T_{r}/7,2T_{r}/7,\ldots ,T_{r}$ . The fitting procedure consisted of minimizing the summed square deviation $\chi^{2}$ between the data and model functions:

30 $$\begin{array}{rl} & \chi_{\text{min}}^{2}:=\underset{r>0,D_{\text{res}}>0}{min}\chi^{2},\text{with}\\ & \chi^{2}:=\sum_{n}\sum_{p=1}^{8}{\left[y_{p}^{\left(n\right)}-F\left(D_{\text{res}},2\pi f_{n},r,t_{p}\right)\right]}^{2}. \end{array}$$

To obtain information about the accuracy of the resulting parameters, the region in the $D_{\text{res}},r$ plane was considered, where $\chi^{2}(D_{\text{res}},r)<2\chi_{\text{min}}^{2}$ .

## 3 Experimental

### 3.1 ${}^{13}C$ solid-state NMR spectroscopy

One part of the ${}^{13}C$ solid-state NMR experiments (spectroscopy, HETCOR, solid-state APT, HMQC) was performed on a Bruker Avance NMR spectrometer operating at 75 and 300 MHz for ${}^{13}C$ and protons, respectively, using a commercial 2.5 mm MAS NMR probe. ${}^{1}H\to {}^{13}C$ CP spectra were recorded with a contact time of 4 ms; 15 000 scans with a recycle delay of 2 s were accumulated. The radio frequency (rf) field strength applied during the CP contact and during the dipolar decoupling corresponds to a nutation frequency of 110 kHz.

To record the directly excited ${}^{13}C$ spectra, 1350 scans were accumulated with a recycle delay of 180 s. The decoupling field strength corresponded to a nutation frequency of 110 kHz.

During the HETCOR experiments, a contact time of 0.7 ms was used: 64 scans with a recycle delay of 2 s were accumulated for each FID, and 450 increments were executed. Ramped ${}^{1}H\to {}^{13}C$ cross-polarization [Bibr bib1.bibx47] and SPINAL-64 proton decoupling [Bibr bib1.bibx13] were applied; the rf field strength corresponded to a nutation frequency of 110 kHz.

To record the solid-state ${}^{1}H$ - ${}^{13}C$ APT spectra, we used for the CP excitation the same parameters as for the CPMAS experiments mentioned above, i.e. contact time 4 ms and rf field strength corresponding to 110 kHz; 20 000 signals were accumulated with a recycle delay of 2 s. ${}^{1}H$ - ${}^{13}C$ through-bond HMQC spectra were obtained with 1600 scans per FID and 70 increments.

DIPSHIFT experiments were performed on a Bruker Avance III spectrometer operating at 100 MHz and 400 MHz for ${}^{13}C$ and protons, respectively, with a commercial 4 mm MAS NMR probe. The spinning speed was varied as described in the Results section. The ${}^{1}H$ nutation frequency was 60.5 kHz, which required an offset of 42.8 kHz for the Lee–Goldburg decoupling within these experiments. To establish the CP, the ${}^{13}C$ rf field strength was set to the effective proton rf field of 74.1 kHz; 3200 to 6400 scans per $t_{1}$ value were recorded with recycle delays of 2 s (25 °C ) and 3 s ( - 15 °C ). The temperature inside the rotor was calibrated using the ${}^{207}\mathrm{Pb}$ chemical shift of lead nitrate as a temperature reference [Bibr bib1.bibx12]. Adamantane served as an external shift standard.

### 3.2 Synthesis of the linker and PIZOF-10

The PIZOFs were obtained through the standard process described for PEPEP-PIZOFs (P stands for phenylene, E for ethynylene) by [Bibr bib1.bibx52]. For the synthesis of PIZOF-10, ${\mathrm{ZrCl}}_{4}$ (0.080 g, 0.34 mmol), ${\mathrm{HO}}_{2}C[\mathrm{PE}\text{-}P(O({\mathrm{CH}}_{2}{\mathrm{CH}}_{2}O{)}_{3}{\mathrm{CH}}_{3}$ , $O({\mathrm{CH}}_{2}{\mathrm{CH}}_{2}O{)}_{3}{\mathrm{CH}}_{3})\text{-}\mathrm{EP}]{\mathrm{CO}}_{2}H$ (0.34 mmol), and benzoic acid (1.256 g, 10.29 mmol) as modulators were dissolved in 20 mL dimethylformamide (DMF). The solution was heated in a tightly capped 100 mL glass flask to 120 °C in an oven for 4 d. The precipitate was isolated from the mother liquor via centrifugation and washed by immersion in DMF for 30 min followed by centrifugation. The immersion and centrifugation steps were repeated with ethanol. The product was dried at reduced pressure, and the obtained dry powder was submitted to Soxhlet extraction with ethanol for 24 h. The mother liquor was again filled into a glass flask and heated to 120 °C . An additional fraction of the product was obtained within 24 h, which was isolated and washed as described for the first crop. All fractions were merged.

## 4 Results

### 4.1 Spectral assignment of the ${}^{13}C$ spectra

The solid-state ${}^{13}C$ NMR spectra of PIZOF-2, PIZOF-10, and PIZOF-11 are well resolved. This allowed us to obtain almost complete ${}^{13}C$ NMR signal assignment for these three samples (see Figs. [Fig Ch1.F5] as well as S1 and S2 in the Supplement together with Table [Table Ch1.T2]). Rough assignment of the solid-state ${}^{13}C$ lines could already be obtained by a comparison to the liquid-state ${}^{13}C$ NMR spectra of the dicarboxylic acid (used for the synthesis of PIZOF-10) in DMSO-d_6_. We observed very similar chemical shifts for most of the detected signals. Significant differences of the order of ca. 5 ppm occur for the carboxylate group (10) and the aromatic carbon (8) directly bound to (10). This is probably caused by the proximity to the metal atom. The signal assignment is further supported by the interpretation of liquid-state DEPT 135 as well as solid-state APT spectra (Fig. [Fig Ch1.F5]). Both methods are able to distinguish differently protonated carbons: in DEPT 135 spectra, the signals of CH and ${\mathrm{CH}}_{3}$ appear positive, whereas ${\mathrm{CH}}_{2}$ groups cause negative resonances. Quaternary carbons do not give any signal in DEPT 135. In analogy to the routine liquid-state APT method, for a certain internal delay, the CH and ${\mathrm{CH}}_{3}$ groups produce negative signals, while the resonances of quaternary carbons and ${\mathrm{CH}}_{2}$ groups are positive. Distinction between quaternary carbons and ${\mathrm{CH}}_{2}$ is also possible based on the significantly higher intensity of quaternary carbon signals compared to the signals of ${\mathrm{CH}}_{2}$ . The signal assignments summarized in Table [Table Ch1.T2] are further corroborated by ${}^{1}H$ - ${}^{13}C$ HETCOR and ${}^{1}H$ - ${}^{13}C$ HMQC spectra: see Fig. [Fig Ch1.F6].

**Figure 5 Ch1.F5:**
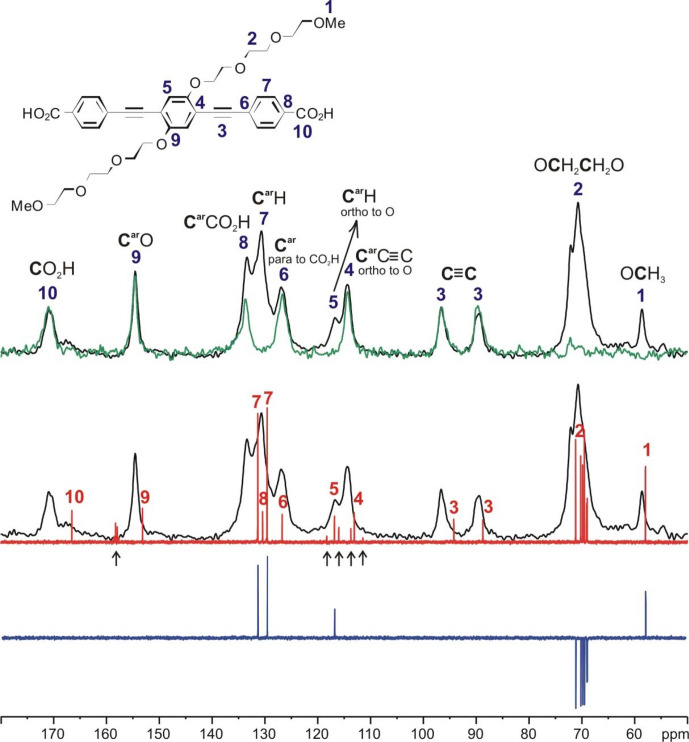
Signal assignment for the ${}^{13}C$ NMR spectrum of the linker of PIZOF-10 based on the interpretation of the ${}^{13}C$ CP MAS NMR spectrum (black), ${}^{13}C$ solid-state APT NMR spectrum (green), ${}^{1}H$ broadband proton-decoupled liquid state (DMSO-d6), ${}^{13}C$ NMR spectrum (red), and liquid-state (DMSO-d6) DEPT 135 ${}^{13}C$ NMR spectrum (blue). The signals marked with ↑ are assigned to trifluoroacetic acid, which was used in the last step of the synthesis of the linkers.

**Table 2 Ch1.T2:** Assignment of the solid-state ${}^{13}C$ NMR signals of PIZOF-10.

Signal	δ (ppm)	Structural group
1	58	${\mathrm{OCH}}_{3}$
2	71	${\mathrm{OCH}}_{2}{\mathrm{CH}}_{2}O$
3	89.5 / 96.5	C≡C
4	115	${\underline{C}}^{\text{ar}}C\equiv C$ (“ortho” to O)
5	117	$C^{\text{ar}}$ (ortho to O)
6	127	$C^{\text{ar}}H$ (“para” to ${\mathrm{CO}}_{2}$ )
7	131	$C^{\text{ar}}H$
8	134	${\underline{C}}^{\text{ar}}{\mathrm{CO}}_{2}H$
9	155	$C^{\text{ar}}O$
10	171	${\mathrm{CO}}_{2}H$

**Figure 6 Ch1.F6:**
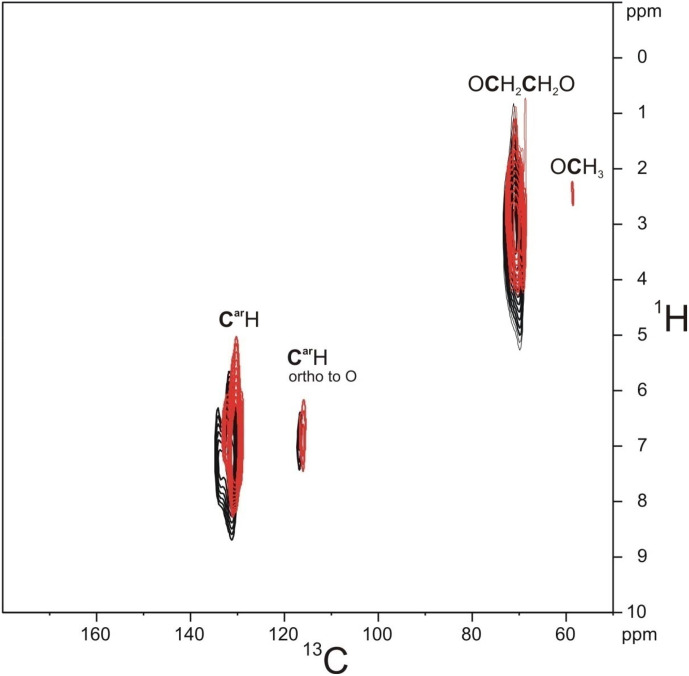
Comparison of the ${}^{1}H$ - ${}^{13}C$ HETCOR spectrum of PIZOF-10 measured at a short contact time (0.7 ms, black) and the ${}^{1}H$ - ${}^{13}C$ HMQC spectrum (red).

Comparing the solid-state ${}^{13}C$ spectra of the different PIZOFs, we found the most relevant differences between the spectra of the different PIZOFs in the ${\mathrm{CH}}_{2}$ region, showing the different kinds of side chains, and in the aromatic region between 111 and 118 ppm. While the PIZOF-2 spectrum simply contains the ${\mathrm{OCH}}_{3}$ resonance, no complete resolution is found for the different aliphatic carbon positions in the chains except for PIZOF-11 at room temperature. Further, the resonance of the CH of the central benzene ring overlaps with the line of ${\underline{C}}^{\text{ar}}C\equiv C$ . It could be separated for PIZOF-10 and PIZOF-11: see below. For PIZOF-2, however, a complete overlap of both resonances occurs, which does not permit a DIPSHIFT analysis of the central-ring CH at all.

### 4.2 Indication of anisotropic motions of the side chains

Figure [Fig Ch1.F7] shows the directly excited ${}^{13}C$ MAS NMR spectra as well as ${}^{13}C$ CP MAS NMR spectra measured with 4 ms contact time for the three samples under study. If the long side chains in PIZOF-10 and PIZOF-11 were liquid-like and would move isotropically, the ${}^{1}H$ - ${}^{13}C$ CP efficiency should be very low or even zero as compared to the ${}^{13}C$ signals of the other framework carbons. The existence of rather strong aliphatic lines implies interpretations either as a consequence of slow motion of the chains, or as indeed fast but spatially restricted reorientation. The latter leads, even for very short correlation times, to a non-zero residual dipolar coupling (see above), which enables cross-polarization even in the case of a fast motion. To distinguish between these cases, the DIPSHIFT method was used.

**Figure 7 Ch1.F7:**
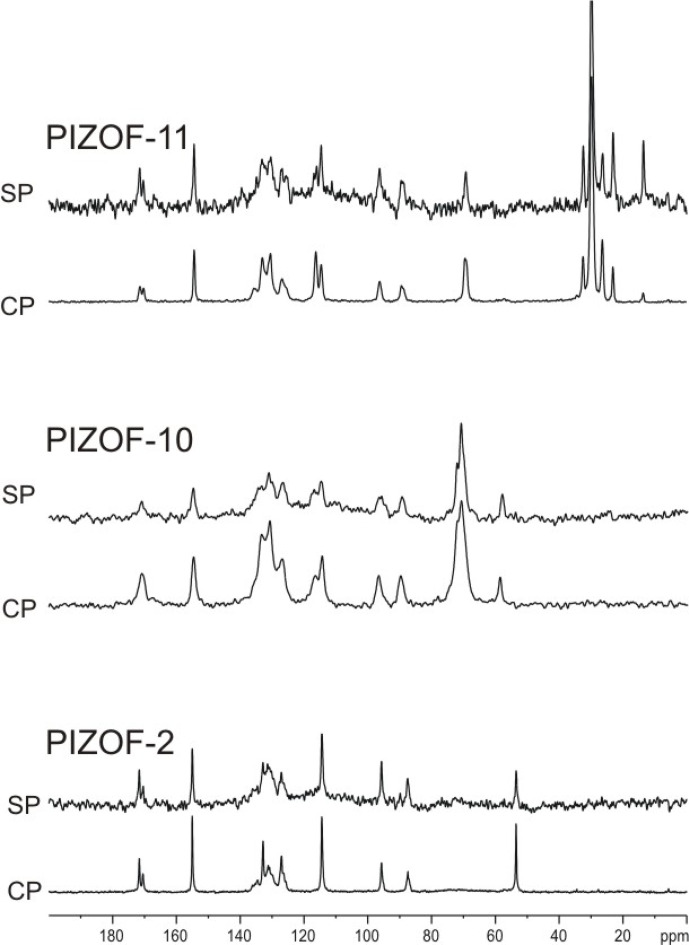
Comparison of directly excited ${}^{13}C$ MAS NMR spectra and ${}^{13}C$ CP MAS NMR spectra (4 ms contact time) of PIZOF-2, PIZOF-10, and PIZOF-11.

### 4.3 Line decompositions

As explained above, we focus on the signals of protonated carbons for the DIPSHIFT data. This concerns the CH of the lateral rings (overlapping at about 130 ppm with the resonances of some quaternary C) and the CH of the central ring. In PIZOF-10 and PIZOF-11, the related lines overlap partially with the line of the quaternary C bound to the acetylenic C (in PIZOF-2 there is a complete overlap which could not be resolved: see above). The side-chain resonances in PIZOF-10 and PIZOF-11 feature some overlap; their resolution could deliver information about the order parameter gradient along the side chain. It turns out that, despite the enhanced uncertainty because of decomposition, well-defined plots of intensity vs. $t_{1}/T_{r}$ were obtained.

Concerning the aliphatic region of PIZOF-11, while at 30 °C five ${\mathrm{CH}}_{2}$ lines are well resolved, at -15 °C there is a strong overlap because of increased line broadening. Figure [Fig Ch1.F9] shows a decomposition in Lorentzian components. The lines could be assigned to the atomic positions by means of the predictions of ACD software [Bibr bib1.bibx1]. Most lines at -15 °C can be assigned immediately to corresponding lines of the 30 °C spectrum, with one exception: the central resonance ( δ position) seems to split at lower temperatures into two overlapping components. The position of one of them corresponds to δ in the 30 °C spectrum, and the other at a distance of about -1.2 ppm is denoted here as $\delta_{1}$ . It is possible that different conformations (e.g. trans and gauche) along the chains are frozen-in at -15 °C , whereas faster motions at 30 °C average them, leading to an average chemical shift.

**Figure 8 Ch1.F8:**
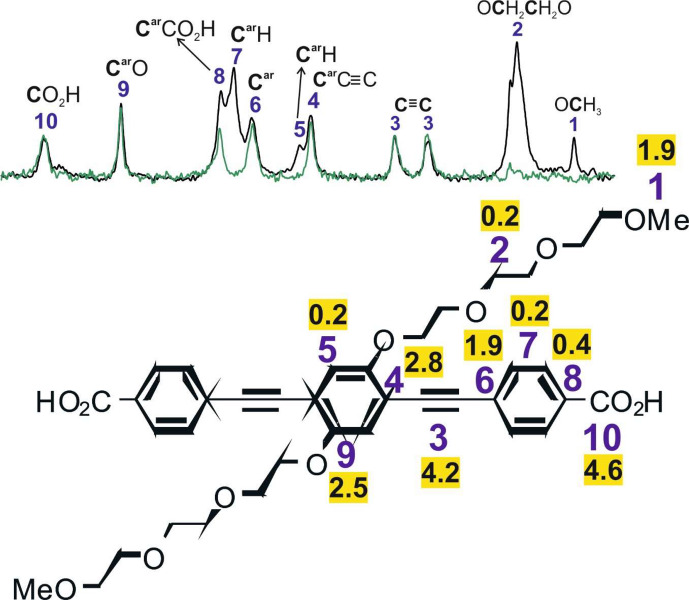
${}^{1}H$ - ${}^{13}C$ CP buildup time constants (ms) for selected ${}^{13}C$ signals of PIZOF-10. In the upper part, the ${}^{13}C$ CPMAS spectrum (black) and the solid-state APT spectrum (green) are shown.

**Figure 9 Ch1.F9:**
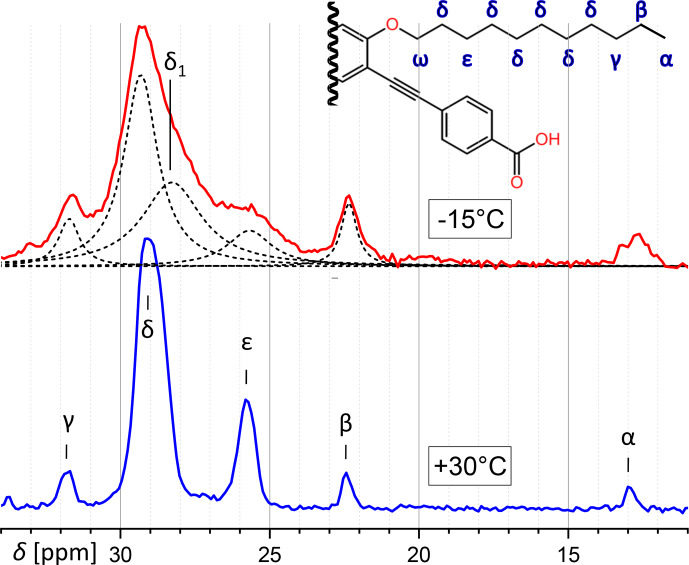
Comparison of the aliphatic regions of ${}^{13}C$ spectra of PIZOF-11 at different temperatures. While at 30 °C different positions are well separated from another, this is not the case at - 15 °C . The decomposition into a number of Lorentzian lines is shown. One component of this decomposition ( $\delta_{1}$ ) seems to have no counterpart at 30 °C . ( ω - ${\mathrm{CH}}_{2}$ is at 69 ppm and is not visible here.)

In some contrast, the signal assignment for the aliphatic carbons (signal 2 in Fig. [Fig Ch1.F5] and in Table [Table Ch1.T2]) of PIZOF-10 is not fully clear. From a comparison with a prediction of isotropic chemical shifts obtained by the ACD software, we expect the resonances of carbons 2b, 2c, 2d, and 2e to be within a region of 0.7 ppm, carbon 2a to be shifted by about 1.2 ppm with respect to the centre of the former group of resonances, and carbon 2f to be shifted by about - 0.6 ppm. (For the sub-numbering of the aliphatic carbons, see Fig. [Fig Ch1.F10].) Taking into account that the solid-state resonances might deviate non-systematically from the solution-based shifts, we could attain a coarse assignment of the - 15 °C lines to the following carbon positions: A to 2a; B to 2b, 2c, 2d, and 2e; and C to 2f. For the five components of the 25 °C spectrum, the assignment will however remain unknown at this stage. Typical solid-state NMR phenomena like conformational differences (e.g. trans or gauche) could generate additional shifts. A possible assignment will be given in the Discussion section.

**Figure 10 Ch1.F10:**
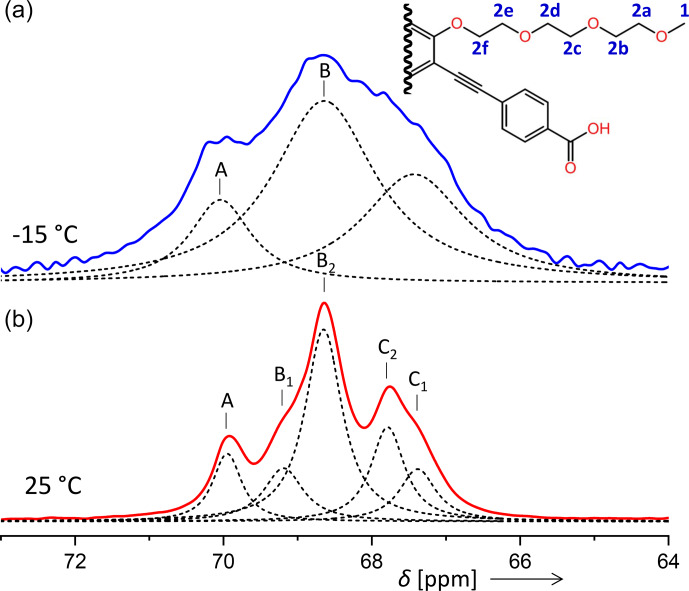
Comparison of the aliphatic regions of ${}^{13}C$ spectra of PIZOF-10 at different temperatures. While at 25 °C five components could be resolved (bottom), at - 15 °C only three were detected (top). The numbering of the carbon positions serves discussions in the subsection “Line decompositions”.

The separation of the ring-CH resonances from overlapping quaternary C resonances is always straightforward. Further line-shape decompositions can be found in the Supplement.

### 4.4 Evaluation of the CP buildup curves

Comparisons of the signal intensities for PIZOF-10 and PIZOF-11 reveal similar CP efficiencies for most side-chain carbon signals to the other ${}^{13}C$ signals of protonated carbons in the framework – except for the chain ends, where the CP efficiency significantly drops (Fig. [Fig Ch1.F7]).

In the CP experiments, several parameters influence the rate and efficiency of polarization transfer, in particular (i) the longitudinal relaxation time of the protons in the rotating frame $T_{1\rho }$ , (ii) the number of neighbouring protons and their distances from the ${}^{13}C$ nuclei, and (iii) the mobility of the nuclei. To investigate the spin polarization transfer, CP buildup curves are measured [Bibr bib1.bibx40]. ${}^{1}H\to {}^{13}C$ CP MAS spectra are recorded with different contact times (here between 0.35 and 10 ms), and the intensities of the individual signals are plotted vs. the contact time. Protons directly bound to the carbon lead to fast and efficient CP buildup and hence to short buildup time constants $T_{\text{CH}}$ , whereas carbons without directly bound protons, e.g. larger distances to protons, show longer CP buildup times. Thermal motions result in a less efficient CP buildup and result in longer CP buildup times $T_{\text{CH}}$ [Bibr bib1.bibx54]. CP buildup curves for PIZOF-10 are shown in the Supplement, and the time constants $T_{\text{CH}}$ obtained from this are shown in Fig. [Fig Ch1.F8] and Table [Table Ch1.T3].

**Table 3 Ch1.T3:** Buildup time constant $T_{\text{CH}}$ values obtained for the ${}^{13}C$ signals in PIZOF-10 (for the corresponding buildup curves, see the Supplement).

Signal	1	2	3	4	5	6	7	8	9	10
Group	${\mathrm{OCH}}_{3}$	${\mathrm{OCH}}_{2}$	C≡C	$C^{\text{ar}}$	$C^{\text{ar}}H$	$C^{\text{ar}}$	$C^{\text{ar}}H$	$C^{\text{ar}}$	$C^{\text{ar}}O$	COOH
$T_{\text{CH}}$ (ms)	1.9	0.2	4.2	2.8	0.2	1.9	0.2	0.4	2.5	4.6

The fastest CP buildups are observed for aromatic CH groups (5, 7) and ${\mathrm{CH}}_{2}$ O groups (2). It is interesting to compare the CP buildup time constants for protonated carbon positions located at the framework (CH groups of the lateral rings 5 and 7) with the time constants for the ${\mathrm{CH}}_{2}$ groups of the side chains (2). If the rigidity of the framework and the side chains were identical, the $T_{\text{CH}}$ of ${\mathrm{CH}}_{2}$ would be shorter than for CH. In our experiments, the values for $T_{\text{CH}}$ of ${\mathrm{CH}}_{2}$ and CH are similar. This indicates that the side chains (2) are slightly more flexible than the framework (5, 7) but are still relatively rigid. Because the signals of the different side-chain positions are not resolved (2), the time constant cannot be determined for the different ${\mathrm{CH}}_{2}$ groups. The DIPSHIFT measurements however allow further interpretations. Theoretically, an immobile ${\mathrm{CH}}_{3}$ group should have the shortest time $T_{\text{CH}}$ compared to all the other carbons in PIZOF-10. The terminal ${\mathrm{CH}}_{3}$ at the end of the side chains (1), however, exhibits a rather long $T_{\text{CH}}$ value. This is likely due to the combined influence of methyl group rotations and the increased mobility of the chain end. It can, therefore, be concluded that the side chains are far from showing a liquid-like behaviour. Obviously, they are relatively ordered and fixed in space due to interactions between themselves and/or the framework. This is in agreement with previous ${}^{13}C$ CP MAS NMR studies, e.g. of n -alkyl silanes bound to silica surfaces [Bibr bib1.bibx56] showing that (i) significant CP transfer occurs for all carbon atoms and (ii) the CP efficiency decreases with increasing distance from the surface, i.e. from the anchoring point of the chain.

### 4.5 Evaluation of the DIPSHIFT curves

DIPSHIFT data and model functions with fitted parameters $D_{\text{res}}$ and r of the methyl carbon in PIZOF-2 are shown in Fig. [Fig Ch1.F11]. DIPSHIFT curves and mean-square deviation surfaces for the other carbon positions which were evaluated can be found in the Supplement.

**Figure 11 Ch1.F11:**
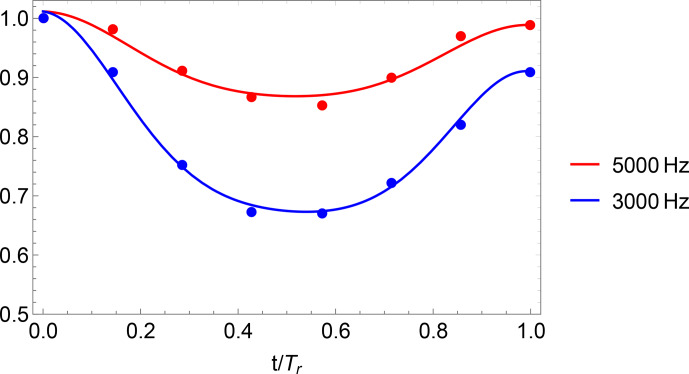
DIPSHIFT curves of ${\mathrm{OCH}}_{3}$ of PIZOF-2 at - 15 °C for different spinning frequencies. The solid lines show model function 4 with values for the parameters $D_{\text{res}}$ and $r_{c}$ which give the best fit to the experimental data for both spinning rates.

Numerical values for $D_{\text{res}}$ and r are summarized in Tables [Table Ch1.T4]–[Table Ch1.T6] for the respective samples. The last columns all contain the order parameters $S=D_{\text{res}}/D_{0}$ . C–H distances are between 1.09 and 1.10 Å, which gives a coupling constant reduced by the Lee–Goldburg scaling factor $D_{\text{LG}}=D_{0}/\sqrt{3}=(13.28\pm 0.18)$ kHz. The resulting uncertainty of about 1.4 % is smaller than the experimental uncertainties and therefore influences the uncertainty of the order parameter only weakly.

**Table 4 Ch1.T4:** Residual dipolar couplings, damping constants, minimum mean-square deviations, and order parameters for methyl carbons and for protonated side-ring carbons in PIZOF-2. For the ring CH, two different fit models were applied: see the text.

Carbon position	Temperature	Fit model	$D_{\text{res}}$ (kHz)	r ( $m\;s^{-1}$ )	$\chi_{\text{min}}^{2}$	S
${\mathrm{CH}}_{3}$	+ 30 °C	4	5.8±0.5	0.4±0.4	0.00395	0.436±0.028
	- 15 °C	4	6.47±0.37	0.68±0.15	0.00147	0.486±0.038
CH (terminal ring)	- 15 °C	1	8.3±1.0	$0.4_{-0.3}^{+0.5}$	0.0516	0.624±0.075
		2	12.6±1.5	–	0.0528	0.947±0.113

**Table 5:** Residual dipolar couplings, damping constants, minimum mean-square deviations, and order parameters for some carbon positions and for components of methylene resonances in PIZOF-10. Again, for the ring CH, two different fit models were applied: see the text.

Carbon position	Temperature	Fit model	$D_{\text{res}}$ /kHz	r ( $m\;s^{-1}$ )	$\chi_{\text{min}}^{2}$	S
${\mathrm{CH}}_{2}$ A	- 15 °C	3	2.9±0.5	$3.3_{-3.3}^{+12}$	0.0225	0.218±0.038
	25 °C	3	2.7±0.6	$1.3_{-1.3}^{+2.6}$	0.00529	0.201±0.045
${\mathrm{CH}}_{2}$ B_1_	25 °C	3	3.4±0.4	$3_{-3}^{+5.6}$	0.00530	0.256±0.030
${\mathrm{CH}}_{2}$ B	- 15 °C	3	3.11±0.25	$0.8_{-0.7}^{+1}$	0.00767	0.234±0.023
${\mathrm{CH}}_{2}$ B_2_	25 °C	3	2.08±0.35	$0.9_{-0.9}^{+6}$	0.0296	0.156±0.038
${\mathrm{CH}}_{2}$ C_2_	25 °C	3	4.64±0.3	$1.4_{-1.2}^{+1.6}$	0.0052	0.349±0.023
${\mathrm{CH}}_{2}$ C	- 15 °C	3	8.2±0.7	$1.1_{-0.8}^{+1.2}$	0.0232	0.614±0.053
${\mathrm{CH}}_{2}$ C_1_	25 °C	3	6.4±0.5	$1.9_{-1.4}^{+2}$	0.0135	0.478±0.038
Middle ring	- 15 °C	1	10.9±1.0	$0.8_{-0.6}^{+1.0}$	0.050	0.823±0.075
		2	16.6±1.5	–	0.051	1.245±0.113
	25 °C	1	10.7±0.9	$0.4_{-0.4}^{+0.8}$	0.0492	0.805±0.068
		2	15.6±2.5	–	0.169	1.173±0.188
Terminal ring	- 15 °C	1	10.2±0.9	$0.5_{-0.5}^{+0.8}$	0.0283	0.767±0.068
	25 °C	1	8.5±0.9	$0.21_{-0.2}^{+0.5}$	0.0637	0.639±0.068
		2	12.8±1.5	–	0.0649	0.962±0.113

**Table 6 Ch1.T6:** Residual dipolar couplings, damping constants, minimum mean-square deviations, and order parameters for some carbon positions in PIZOF-11. Again, for the ring CH, two different fit models were applied: see the text.

Line assignment	Temperature	Fit model	$D_{\text{res}}$ (kHz)	r ( $m\;s^{-1}$ )	$\chi_{\text{min}}^{2}$	S
β - ${\mathrm{CH}}_{2}$	- 15 °C	3	$1.5_{-0.5}^{+0.3}$	$0.5_{-0.5}^{+4}$	0.0389	$0.11_{-0.04}^{+0.024}$
	25 °C	3	1.0±0.5	$6.4_{-6.0}^{+50}$	0.0128	$0.075_{-0.045}^{+0.030}$
	30 °C	3	$1.2_{-0.4}^{+0.3}$	$0.3_{-0.3}^{+3.5}$	0.0238	$0.09_{-0.03}^{+0.02}$
γ - ${\mathrm{CH}}_{2}$	- 15 °C	3	1.88±0.5	$0.50_{-0.5}^{+3.3}$	0.0630	0.14±0.04
	25 °C	3	1.28±0.3	$3.6_{-3.6}^{+6}$	0.0097	0.10±0.03
	30 °C	3	1.6±0.4	$0.3_{-0.3}^{+2}$	0.0324	$0.12_{-0.03}^{+0.02}$
δ - ${\mathrm{CH}}_{2}$	- 15 °C	3	2.5±0.4	$0.5_{-0.5}^{+1.1}$	0.0347	0.19±0.03
	- 15 °C	3	5.0±0.7	$0.23_{-0.1}^{+0.2}$	0.0367	0.37±0.06
	25 °C	3	1.94±0.16	$1.4_{-0.5}^{+0.8}$	0.0048	0.15±0.01
	30 °C	3	2.33±0.25	$0.36_{-0.25}^{0.4}$	0.0246	0.17±0.02
ε - ${\mathrm{CH}}_{2}$	- 15 °C	3	3.5±0.5	$0.31_{-0.3}^{+0.8}$	0.0374	0.26±0.034
	25 °C	3	2.51±0.11	0.48±0.25	0.0030	0.19±0.01
	30 °C	3	2.44±0.2	$0.34_{-0.3}^{+0.5}$	0.0129	0.18±0.015
ω - ${\mathrm{CH}}_{2}$	- 15 °C	3	7.1±0.9	$0.8_{-0.5}^{+0.9}$	0.1040	0.53±0.068
	25 °C	3	5.5±0.3	0.40±0.16	0.0150	0.41±0.02
	30 °C	3	5.55±0.45	$0.22_{-0.09}^{+0.13}$	0.0247	0.42±0.03
Middle ring	- 15 °C	1	$9.9_{-0.9}^{+1.1}$	$0.13_{-0.13}^{+0.9}$	0.1250	$0.74_{-0.07}^{+0.090}$
		2	14.4±3.1	–	0.1610	1.1±0.02
	25 °C	1	10.0±0.4	0.34±0.3	0.0053	0.76±0.03
		2	15.2±0.6	–	0.0055	1.14±0.05
	30 °C	1	9.1±1.1	$0.16_{-0.16}^{+0.64}$	0.0567	0.68±0.08
		2	13.7±1.8	–	0.0580	1.03±0.14
Terminal ring	- 15 °C	1	9.9±1.9	$0.13_{-0.13}^{+1.9}$	0.1250	0.75±0.14
	25 °C	1	$9.4_{-1.1}^{+1.25}$	$0.066_{-0.066}^{+0.3}$	0.1530	0.71±0.09
	30 °C	1	10.8±1.3	$0.27_{-0.27}^{+0.55}$	0.0729	0.80±0.10

The accuracy of the order parameter values can also be affected by the inhomogeneity of the RF field. The latter leads to an inhomogeneity of the Lee–Goldburg scaling factor (LG factor; Lee and Goldburg, 1965), which is important for determining the order parameter. However, it was possible to estimate that the resulting measurement uncertainty is less than 1 %. For this estimation, data from Wurl et al. (2023) were used (see the Supplement).

## 5 Discussion

The evolution of the ${}^{13}C$ magnetization during a ${}^{1}H$ - ${}^{13}C$ cross-polarization experiment is characterized for short times ( $∼D_{\text{res}}^{-1}$ ) by powder-averaged oscillations determined by the (residual) dipolar coupling. For longer times, however, spin diffusion within the proton system will be more and more important; it provides a re-polarization of those protons which are close to ${}^{13}C$ and have been depolarized during CP. Both phenomena are expected to be slowed down by thermal motion.

The $T_{\text{CH}}$ values corresponding to the long-time buildup indicate an increased mobility towards the end of the side chains. With regards to the related thermal motion, we can distinguish two cases: (i) slow or intermediate nearly isotropic motion, which would cause a loss of CP efficiency upon speeding up, and (ii) fast anisotropic motion, which does not hamper CP buildup even in the fast limit because of the residual dipolar coupling. The latter enables both oscillatory behaviour in the CH systems at short times and spin diffusion in the proton system at long times. The DIPSHIFT results provide a distinction between these two options. The first case would lead to strongly damped rotational echoes in the FID; see above. A re-increase in the second half of the DIPSHIFT curve would not occur. The DIPSHIFT signals obtained for the PIZOF samples, however, show a clear recovery of the rotational echo, almost reaching the initial FID intensity.

In most of the cases investigated here, the residual dipolar couplings are essentially smaller than the value which would occur for immobile structural groups. Consequently, thermal motion that effectively averages the dipolar interactions must be present. This motion must be much faster than the strength of carbon–proton dipolar coupling. In other words, the correlation times must be shorter than $10^{-5}$ s (in the intermediate region in a range $10^{-5}\ldots 10^{-3}$ s, the motion would lead to a strong damping of the rotational echoes). The rotational echo after one rotation period is slightly damped but in no case to less than 70 % of the initial signal. This means that fast motion reduces the apparent dipolar coupling and the existence of a rather slow motion with correlation times of $2\times 10^{-3}$ s causing the observed weak damping of the rotational echoes for room temperature as well as for -15 °C . This slow motion leads to further averaging of the dipolar interaction but just at times which are much longer than the duration of the DIPSHIFT modulation period.

The side-chain order parameter could be estimated for different positions on the basis of our line decomposition, remembering that only for PIZOF-11 at 25 °C are the aliphatic lines well resolved. Besides the $\delta_{1}$ component occurring at - 15 °C only (see Fig. [Fig Ch1.F9]), all the low-temperature components of the deconvolution could be assigned to the well-separated room temperature lines and therefore also to carbon positions. A gradient of the order parameter along the chain could be detected: see Fig. [Fig Ch1.F12]. On this basis, we can give a possible explanation for the occurrence of the $\delta_{1}$ component. The value of its order parameter, which is placed between the S values of ε carbons and ω carbons, implies an assignment to the carbon being placed next to the ω carbon. At room temperature, its resonance is part of the signal group of the δ carbons. Perhaps because of conformational effects, it is shifted towards higher parts-per-million values at - 15 °C .

**Figure 12 Ch1.F12:**
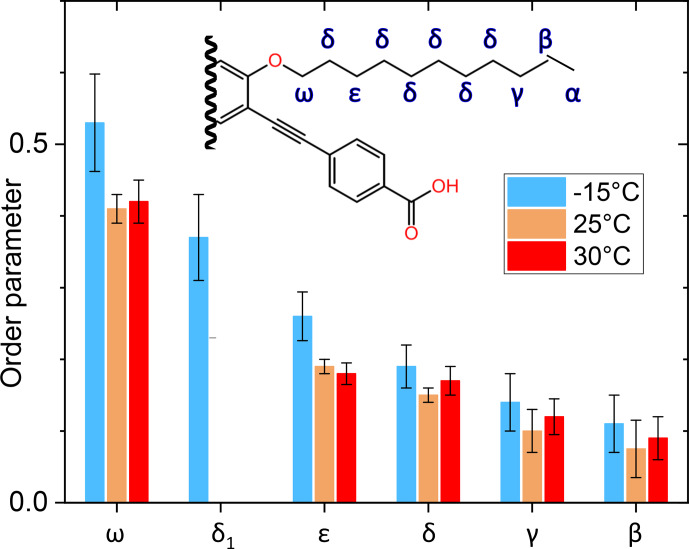
Graphical representation of the order parameter gradient along the side chains of PIZOF-11. The label “ $\delta_{1}$ ” marks the position in the chain where the origin of this line is assumed; see the text.

The order parameters of the side chains in PIZOF-10 are shown in Fig. [Fig Ch1.F13]. Following the liquid-state assignment (see “Line decompositions”), component A was assigned to position 2a and component C was assigned to position 2f. The order parameter of the carbon at position 2f is clearly larger than that of the remaining part of the side chain. This phenomenon also occurring in PIZOF-11 can be understood by the proximity of these carbons to the more rigid central rings: see below. The value of the order parameter of component C_2_ strongly suggests an assignment of this component to position 2e. The order parameter of component B_1_ exceeds that of B_2_ only weakly but is beyond the limits of uncertainty. Hence, an assignment of this component to carbon position 2d is likely to some degree.

A comparison of the different side-chain groups linked via O to the central rings shows that the order parameters of PIZOF-10 and PIZOF-11 are equal within experimental uncertainty. However, in PIZOF-2 at -15 °C , the bond-order parameter of the methyl group is clearly smaller than that of the ω - ${\mathrm{CH}}_{2}$ of both other PIZOFs, remembering that we have considered the fast rotation around the methyl symmetry axis in the fit. This indicates that there is a fast motion of the methyl axis itself which takes place in a range of angles which is of a larger amplitude than in the case of the ω methylenes in PIZOF-10 and PIZOF-11.

**Figure 13 Ch1.F13:**
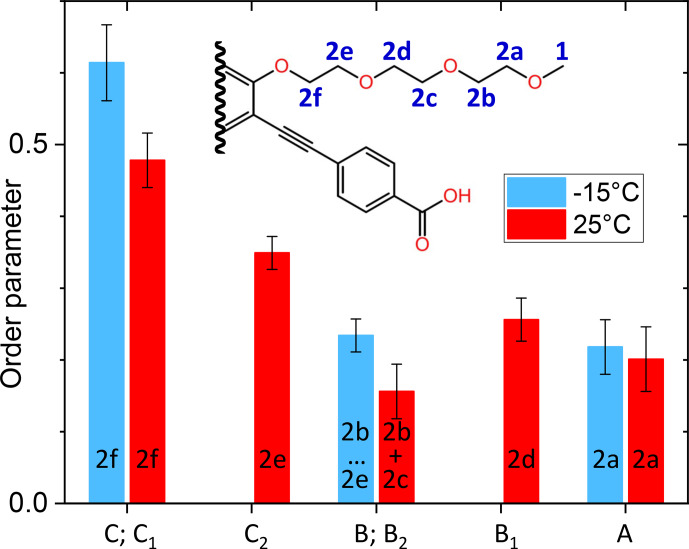
Graphical representation of the order parameters for the different components of the methylene line in PIZOF-10.

The order parameters of the central rings are graphically compared in Fig. [Fig Ch1.F14]. The PIZOF-2 spectrum did not allow for a separation of the corresponding line from a completely overlapped group of signals. Hence, we now only deal with the two other samples. For PIZOF-10, similar order parameters (0.74 and 0.68 for - 15 and 30 °C , respectively) to PIZOF-11 (0.82 and 0.80 for the same temperatures) were determined. We observe that the first ones are slightly smaller but are not beyond the limits of experimental uncertainty. The 180° flips of the rings are not expected because of the linked side chains. Indeed, an attempt to evaluate the DIPSHIFT curves by model function 2 (for 120° jumps of the CH bonds) failed; it delivered unrealistically high values for the residual dipolar coupling.

**Figure 14 Ch1.F14:**
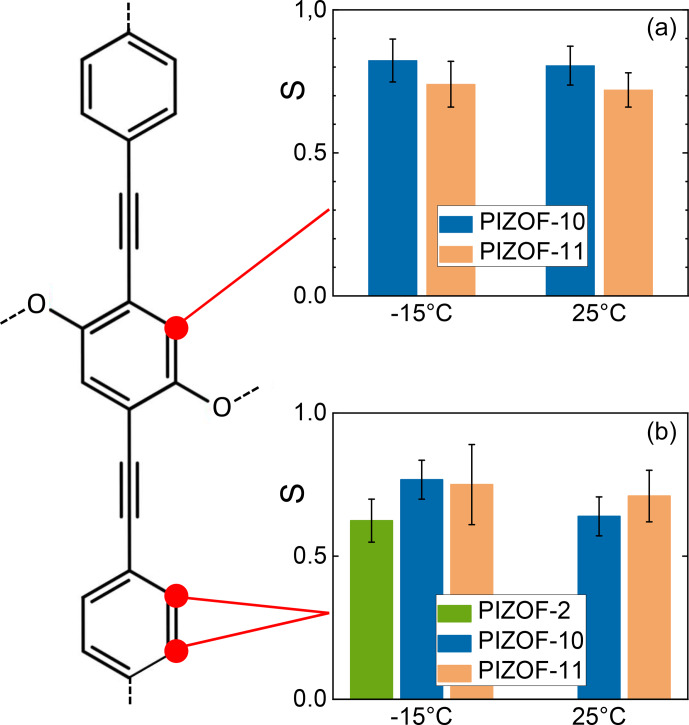
Graphical representation of the order parameters for the protonated carbons in the middle rings **(a)** and terminal rings **(b)**.

With regards to the terminal rings, flips could also not be detected; the fit attempt led to $D_{\text{res}}$ values which would be larger than those expected for rigid molecular parts. Evidence of such rapid motion would only be given if $D_{\text{res}}<D_{\text{LG}}$ had been found to be outside the error limits. This also concerns slow flips, as this would lead to a larger $T_{2}$ damping of the rotational echo. An order parameter <1 thus has to be explained by fast small-angle motions. For the central ring, π flips have a very small likelihood because of the attached side chains.

## 6 Conclusions

Long aliphatic and ethyleneglycol side chains of the PIZOF samples were shown to exhibit a gradient of anisotropy of motion. Not only the terminal methyl groups, but also the methylene groups close to the ${\mathrm{CH}}_{3}$ are subject to fewer restrictions than the inner ${\mathrm{CH}}_{2}$ groups. The carbons closest to the linker backbone are subject to the strongest motional restrictions.

“Restriction” in our sense does not concern the speed of motion but the degree of anisotropy. Assuming that this anisotropic motion would proceed as fluctuations of the ${\mathrm{CH}}_{2}$ plane normals or ${\mathrm{CH}}_{3}$ rotation axes within a cone with a defined opening angle, this angle would be larger the smaller the order parameter is. This is reminiscent of the dynamics in nematic liquid crystals; overall, we observe orientational restrictions to different degrees despite rather short correlation times. The difference in liquid crystals is found in the existence of the long-range order of the chains of the latter.

### 6.1 Cos-Fourier coefficients

#### 6.1.1 Case 1: ensemble of rigid IS spin pairs

C1IS=215D0ωr2-171260D0ωr4+131205920D0ωr6-12583705729024D0ωr8+3569811072708116480D0ωr1031-252701875693604618240000D0ωr12

C2IS=160D0ωr2+1720D0ωr4-298323063040D0ωr6+6386314114580480D0ωr8-2688432860554977280D0ωr1032+5635245142295348592640000D0ωr12

C3IS=11260D0ωr4-3978648640D0ωr6+353235243008D0ωr8-17669536354058240D0ωr1033+6015291174870794240000D0ωr12

#### 6.1.2 Case 2: spin pairs undergoing fast two-site jumps

See Eq. ([Disp-formula Ch1.E9]).

C0IS-j=1-21320D0ωr2+1589737280D0ωr4-188721747233105920D0ωr6+1111884723492397367296D0ωr8-757129037194773330899763200D0ωr1034+9126896358637389583325014754590720000D0ωr12

C1IS-j=7120D0ωr2-11946080D0ωr4+3219835904138240D0ωr6-46015216607236759552D0ωr8+19314981732137599598460928D0ωr1035-465893115772791241796848484530257920000D0ωr12

C2IS-j=7960D0ωr2+49184320D0ωr4-25705718893242368D0ωr6+32474753132144735191040D0ωr8-1107477091428501327979479040D0ωr1036+912767511822134967187393938121031680000D0ωr12

C3IS-j=746080D0ωr4-141237084965888D0ωr6-7673311012061265920D0ωr8+3009981180343998996152320D0ωr1037-58741942147313797742760939225088ß0000D0ωr12

#### 6.1.3 Case 3: spin triples IS_2_

C0IS2=1-310D0ωr2+2274536D0ωr4-494719729720D0ωr6+293281840647808D0ωr8-28631116716662021667200D0ωr1038+142610164787225135039577536000D0ωr12

C1IS2=415D0ωr2-34567D0ωr4+2843405405D0ωr6-1768933776028D0ωr8+227257983310108336D0ωr1039-258076147246858596028000D0ωr12

C2IS2=130D0ωr2+1162D0ωr4-24371297296D0ωr6+9662394728643920D0ωr8-2084551158685920640D0ωr1040+687435652711938979371536000D0ωr12

C3IS2=2567D0ωr4-2291459458D0ωr6-9379394053660D0ωr8+22469969425090280D0ωr1041-1930764389998496579815172000D0ωr12

#### 6.1.4 Case 4: spin quartets IS_3_

C0IS3=1-120D0ωr2+22777760D0ωr4-67283560431872D0ωr6+125038733919820636160D0ωr8-1224110592116703143526400D0ωr1042+48124765715776357267735755489280000D0ωr12

C1IS3=245D0ωr2-174860D0ωr4+799150038560D0ωr6-68829011543429375488D0ωr8+13208297158752735764480D0ωr1043-11191407716691008604977307361280000D0ωr12

C2IS3=1180D0ωr2+719440D0ωr4-1819635604318720D0ωr6+3493306130868587509760D0ωr8-9947191423340628705280D0ωr1044+24956809974377492494117140398080000D0ωr12

C3IS3=14860D0ωr4-242172101619520D0ωr6+193091514476458496D0ωr8-65375379376367882240D0ωr1045+26639914823208124836587233280000D0ωr12

## Supplement

10.5194/mr-5-1-2024-supplement The supplement related to this article is available online at https://doi.org/10.5194/mr-5-1-2024-supplement.

## Supplement

10.5194/mr-5-1-2024-supplement
10.5194/mr-5-1-2024-supplement The supplement related to this article is available online at https://doi.org/10.5194/mr-5-1-2024-supplement.

## Data Availability

The data sets generated and analyzed for this study as they appear in the figures of this article can be found in the Zenodo repository: 10.5281/zenodo.10156265 [Bibr bib1.bibx18].

## Appendix

The supplement related to this article is available online at https://doi.org/10.5194/mr-5-1-2024-supplement.
