# Supplementary material for: Solid-state 13C-NMR spectroscopic determination of side-chain mobilities in zirconium-based metal–organic frameworks
Source: Magn Reson (Gott). 2024 Jan 5;5(1):1–20. doi: 10.5194/mr-5-1-2024 (PMC12082566; doi:10.5194/mr-5-1-2024)
Supplement: The supplement related to this article is available online at https://doi.org/10.5194/mr-5-1-2024-supplement. [file mr-5-1-2024-supplement.pdf]

Supplement of Magn. Reson., 5, 1–20, 2024
https://doi.org/10.5194/mr-5-1-2024-supplement
© Author(s) 2024. CC BY 4.0 License.

Supplement of

Solid-state ^{13}C-NMR spectroscopic determination of side-chain mobilities in zirconium-based metal–organic frameworks

Günter Hempel et al.

Correspondence to: Günter Hempel (guenter.hempel@physik.uni-halle.de)

The copyright of individual parts of the supplement might differ from the article licence.

Pulse	Phase cycle
First proton pulse	$x\bar{x}$
CP: ^{1}H	y
CP: ^{13}C	$xxyy\ \bar{x}\bar{x}\bar{y}\bar{y}$
^{13}C refocusing π pulse	$xxyy\ \bar{x}\bar{x}\bar{y}\bar{y}\ yy\bar{x}\bar{x}\ \bar{y}\bar{y}xx$
Receiver	$x\bar{x}y\bar{y}\ \bar{x}x\bar{y}y\ \bar{x}x\bar{y}y\ x\bar{x}y\bar{y}$

Table I. Phase cycle for the DIPSHIFT pulse sequence as applied in the experiments shown in this paper..

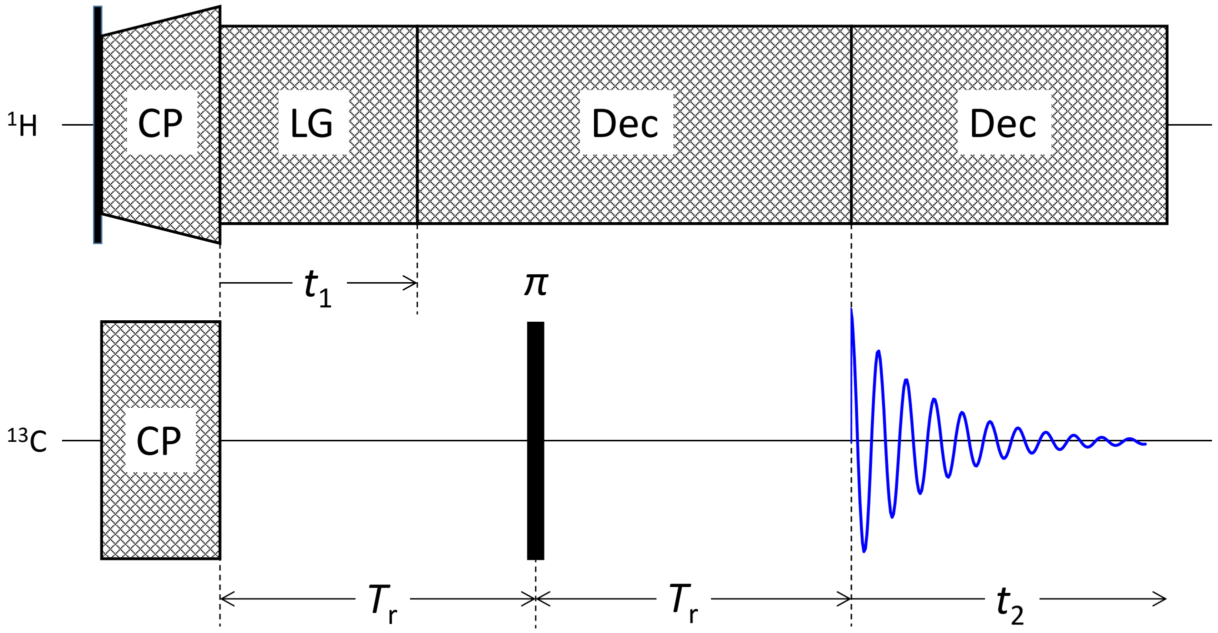

Figure S1. Pulse scheme for the DIPSHIFT experiment. Meaning of the abbreviations: CP = cross polarization, LG = irradiation under Lee-Goldburg condition, Dec = decoupling in resonance. t_1 is the variable for the indirect dimension and will be incremented from experiment to experiment between 0 and T_r. T_r is the rotational period.

S1. EXPERIMENTAL SCHEME

The pulse sequence is shown in Fig. S1; the phase cycle can be found in table I.

S2. LINE DECOMPOSITIONS

See Figs. S2, S3, and S4.

S3. CP BUILDUP CURVES

For Pizof-10, the CP builup curves are shown in Fig. S5.

S4. DIPSHIFT DIAGRAMS

In the diagrams in this section (Figs. S7 - S28), at the left-hand side the data are shown together with the fitted model functions. The right-hand sides show plots of the mean-square deviation χ^2 versus damping constant r and residual dipolar coupling D_{res}. In the latter, the cross marks the position of minimum mean-square deviation χ^2_{min}; the closed line connects all points where $\chi^2 = 2\chi^2_{\text{min}}$. The evaluation was not executed for all spinning frequencies; it was omitted for such frequencies where the line under consideration had a distance of an integer multiple of spinning frequency to another line.

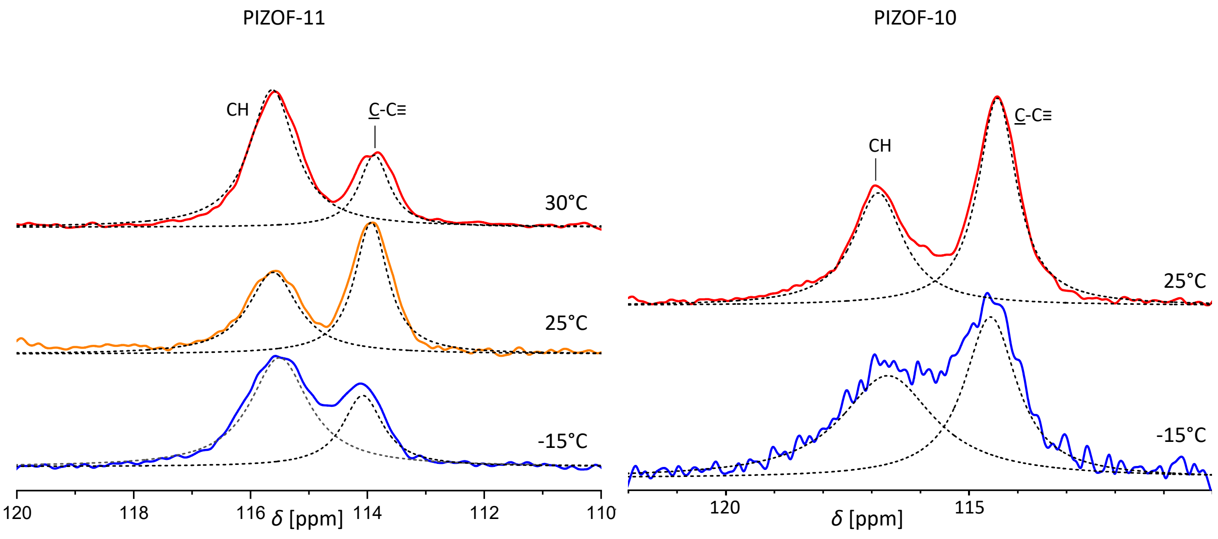

Figure S2. Decomposition of overlapping middle-ring lines of both protonated carbons and those carbons bound to C≡C in PIZOF-10 (right) and in PIZOF-11 (left).

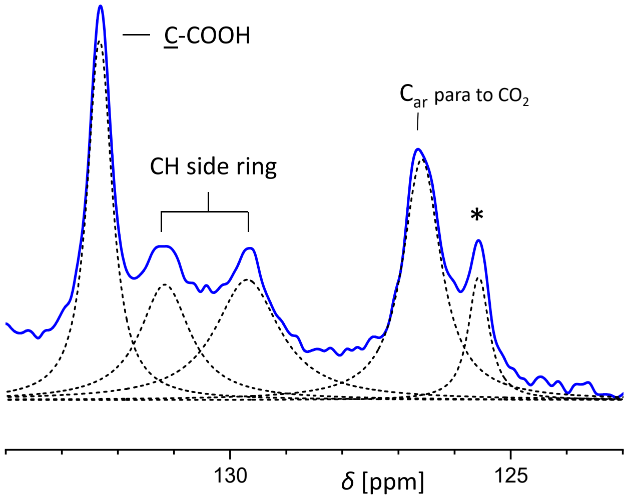

Figure S3. Decomposition of overlapping lines in the region 120 ... 140 ppm in PIZOF-2 Spinning sidebands are marked by asterisks.

S5. INFLUENCE OF B_1 INHOMOGENEITY ON THE LG SCALING FACTOR

For this estimate, B_1 data from Wurl et al. (2023) are used. These were determined on the same probe with which the DIPSHIFT measurements shown here were carried out. The authors there were able to approximate the spatial distribution of B_1 along the coil axis with a Gaussian function. Since the relevant range for the sample ends where B_1 is still more than approximately 50 % of the maximum value, it is sufficient for this estimation, to use the field curve as a Taylor expansion up to the quadratic term:

$$B_1(z) = B_{1\max} - 4\Delta B_1 \, \frac{z^2}{L^2} \tag{1}$$

z is the coordinate along the axis of the rf coil; its origin is in the middle of the coil. $B_{1\max}$ is the maximum rf field. The length of the sample is denoted by L. At the edges of the sample (positions $+L/2$ and $-L/2$), the field strength is $B_1 - \Delta B_1$ according to this approximation. ΔB_1 then characterizes the strength of the rf inhomogeneity.

The arithmetic mean over the sample is

$$\langle B_1 \rangle = \frac{1}{L}\int_{-L/2}^{L/2} B_1(z)\,\mathrm{d}z = B_{1\max} - \frac{1}{3}\Delta B_1 \tag{2}$$

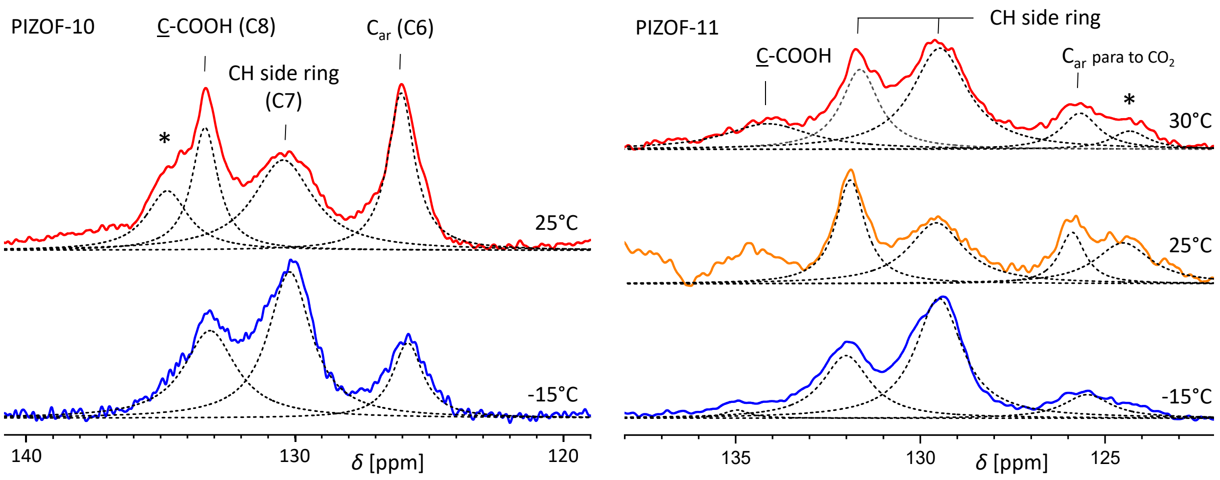

Figure S4. Decomposition of overlapping lines in the region 120 ... 140 ppm in PIZOF-10 and in PIZOF-11. For Pizof-10, the numbering of the carbon positions corresponding to Fig. 5 and table 1 are given. Spinning sidebands are marked by asterisks.

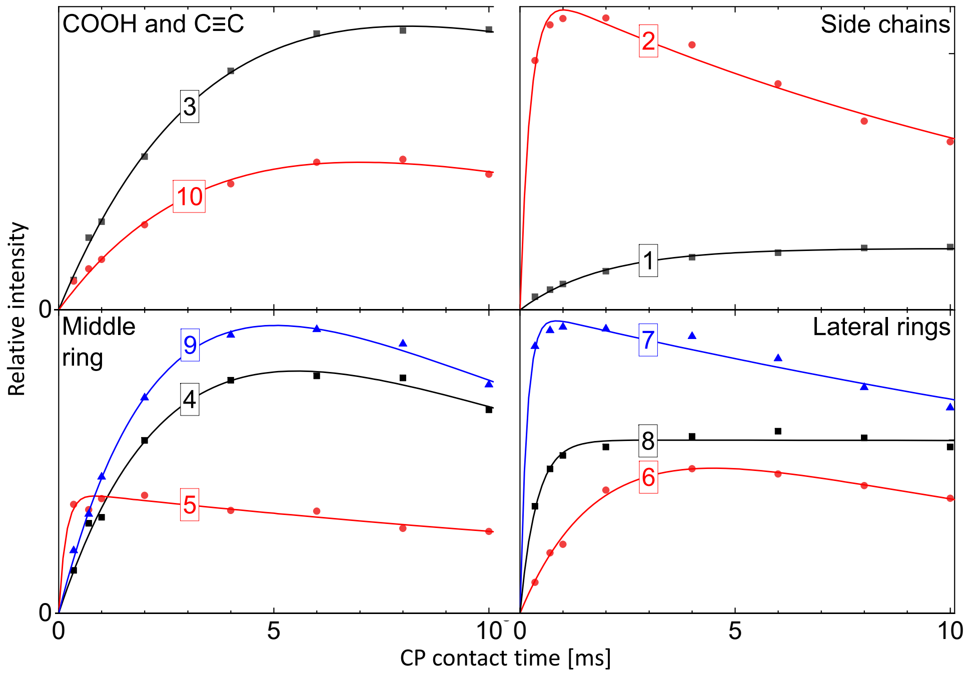

Figure S5. CP buildup curves of Pizof-10. The numbering corresponds to the different atomic positions in fig. 5 and table 2 (main part).

This mean value has been obtained by determining B_1 from the zero crossing of the transverse magnetization near the 180° condition. It is used to determine the value of the deviation between resonance and irradiation $\Delta B = B_1\sqrt{2}$ which is needed for eliminating the proton-proton dipolar interaction. This results in a scaling factor ε for the dipolar interaction between protons and ^{13}C spins

$$\varepsilon^2 = \frac{\Delta B^2}{\Delta B^2 + B_1^2} \tag{3}$$

which has the value $\varepsilon = 1/\sqrt{3}$ if the adjustment is exact. An inhomogeneous rf field leads to a spatial dependence of ε. The polynomial equations which describe the shape of the DIPSHIFT curves (section 7 of the main part) contain at the leading positions terms which are proportional to $D^2_{\rm res}$. That means

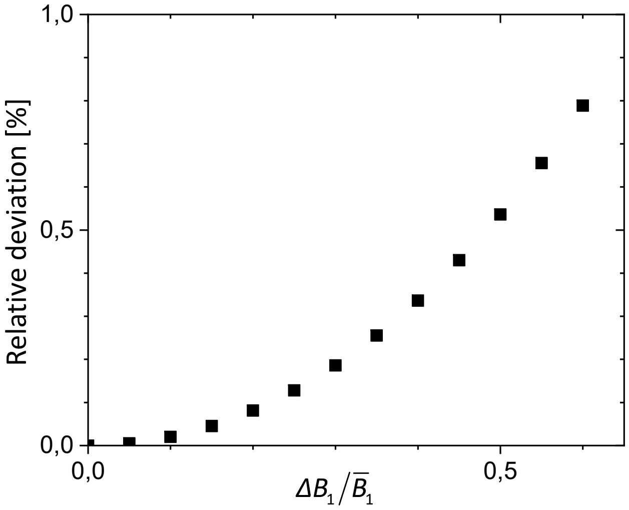

Figure S6. Relative deviation $(\sqrt{\langle\varepsilon^2\rangle} - 1/\sqrt{3}) \cdot \sqrt{3}$ of the mean-square LG scaling factor from its ideal value vs. the relative rf field inhomogeneity.

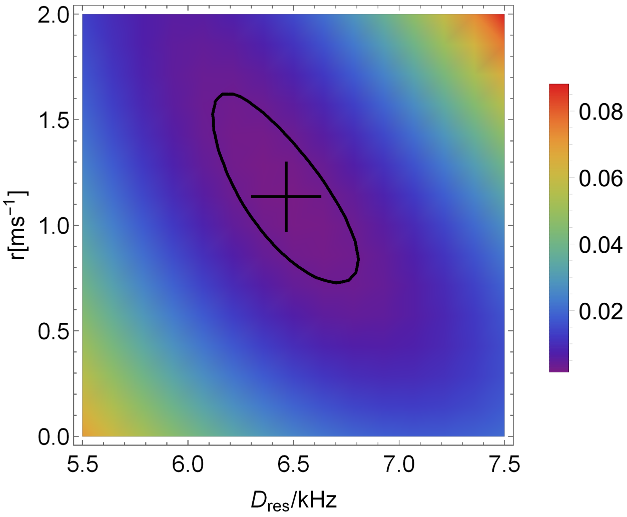

Figure S7. To Fig. 11 in the main part: Mean square deviation χ^2 between model function 4 and experimental DIPSHIFT data of CH_3 of PIZOF-2 vs. varied parameters D_{res} and damping constant r. The cross marks the position of minimum χ^2; the closed line marks the region where $\chi^2(D_{\mathrm{res}}, r) = 2\chi^2_{\mathrm{min}}$. For a Gaussian distribution this would be the distance of one standard deviation from the minimum.

that for our estimation of uncertainty of DIPSHIFT curves due to rf inhomogeneity, the mean square of the scaling factor is relevant. We obtain it by inserting equations (1) and (2) into equation (3) and integration with substitution $z/L \to z_1$:

$$\langle\varepsilon^2\rangle = \frac{1}{L}\int_{-L/2}^{L/2} \frac{\Delta B^2}{\Delta B^2 + \left(\langle B_1\rangle + \frac{\Delta B_1}{3} - 4\Delta B_1 \frac{z^2}{L^2}\right)^2} \,\mathrm{d}z = \int_{-1/2}^{1/2} \frac{\mathrm{d}z_1}{1 + 2\left(1 + \frac{1}{3}\frac{\Delta B_1}{\langle B_1\rangle} - 4\frac{\Delta B_1}{\langle B_1\rangle} z_1^2\right)^2} \tag{4}$$

For an homogeneous B_1 (i.e. $\Delta B_1 = 0$) we obtain the value 1/3 if the LG condition is adjusted. Fig. S6 shows the result of a numerical evaluation of equation (4) vs. strength of inhomogeneity. The relative deviation of $\sqrt{\langle\varepsilon^2\rangle}$ from its ideal value $1/\sqrt{3}$ is plotted versus $\Delta B_1/\langle B_1\rangle$. It can be concluded that even quite strong field inhomogeneities leave the deviation from the ideal value at less than 1 %.

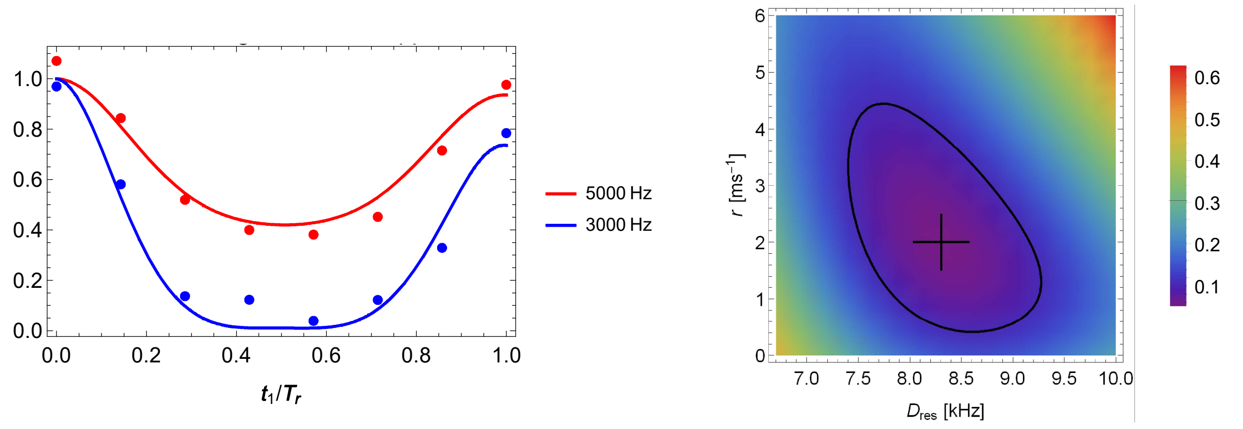

Figure S8. PIZOF-2, -15°C, CH of side ring, model function 1.

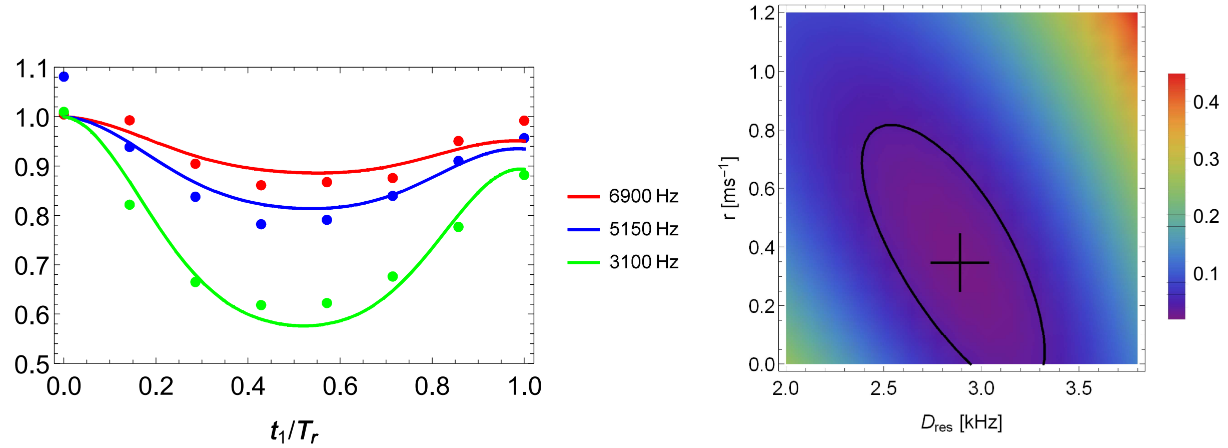

Figure S9. PIZOF-10, -15°C, CH_2, left component, model function 3.

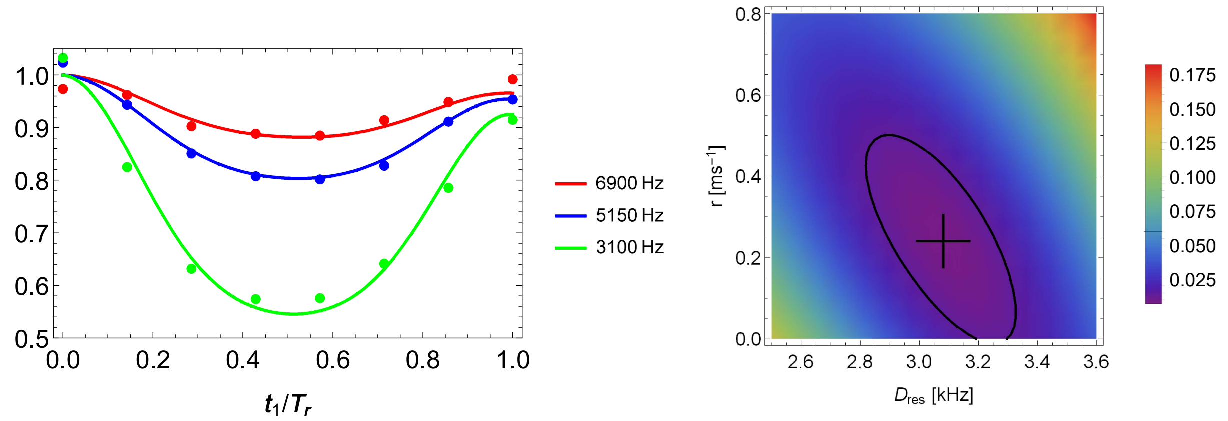

Figure S10. PIZOF-10, -15°C, CH_2, middle component, model function 3.

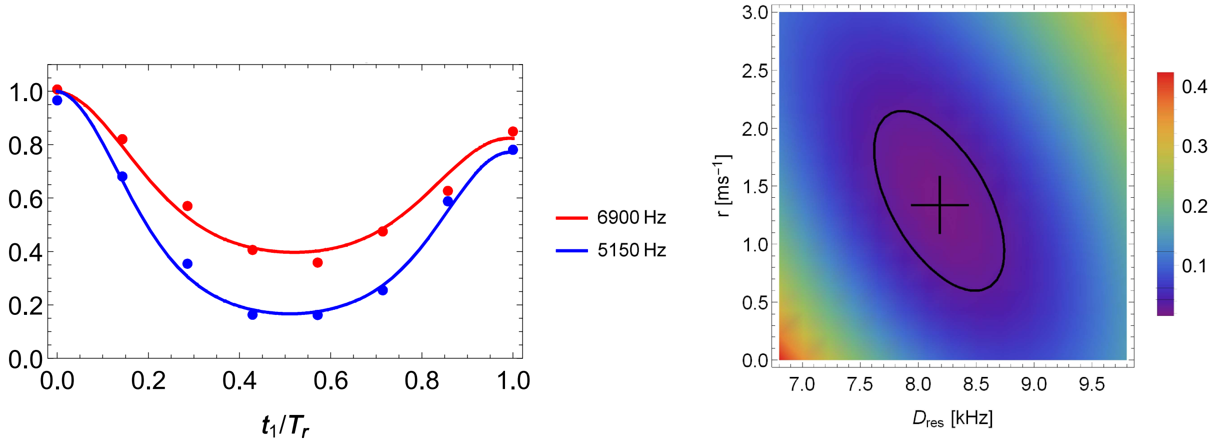

Figure S11. PIZOF-10, -15°C, CH_2, right component, model function 3.

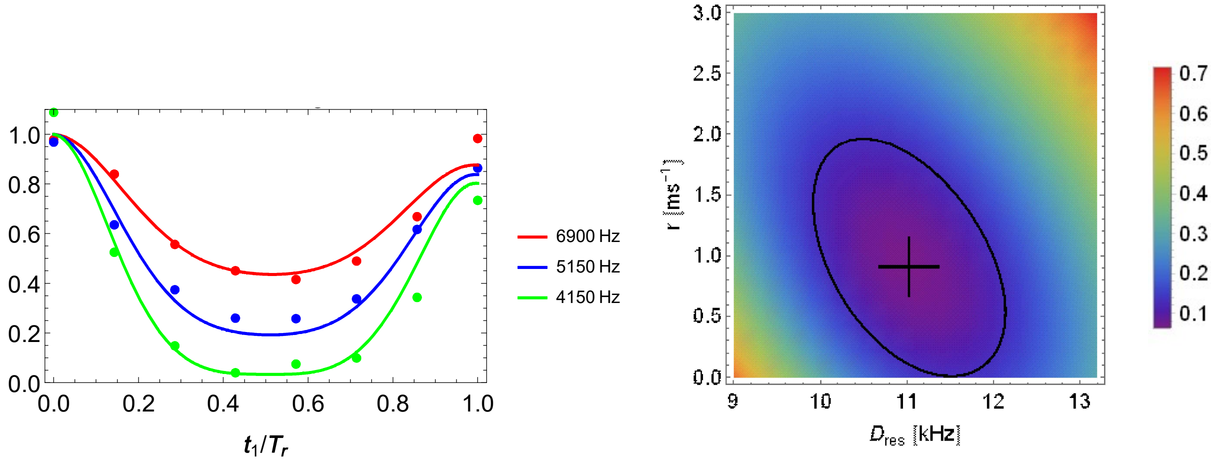

Figure S12. PIZOF-10, -15°C, CH of side ring, model function 1.

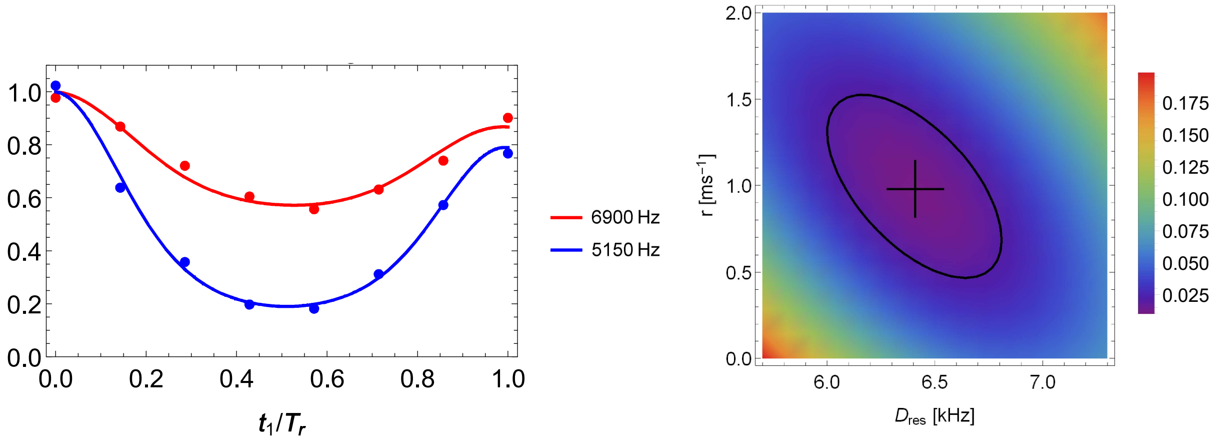

Figure S13. PIZOF-10, 25°C, CH_2 right component, model function 3.

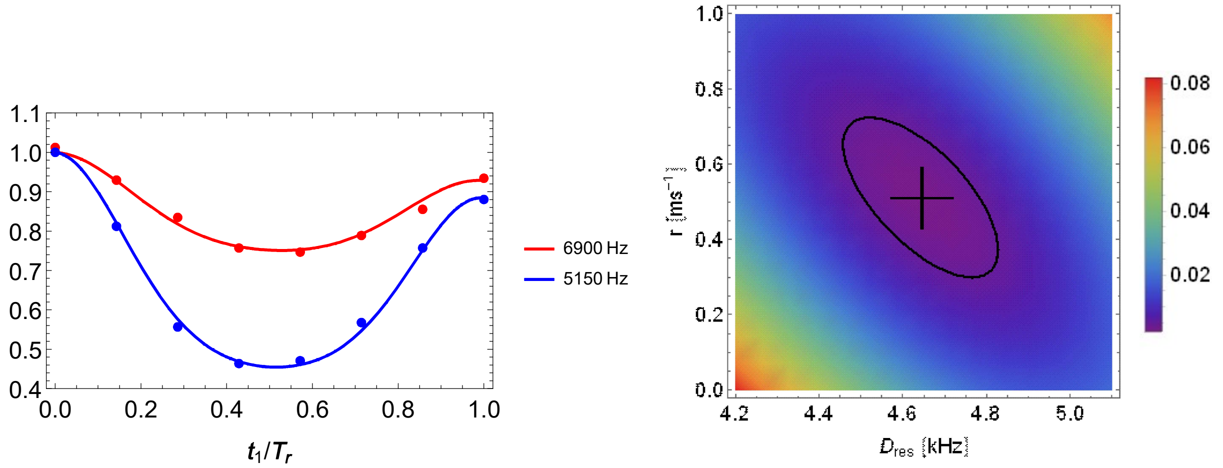

Figure S14. PIZOF-10, 25°C, CH_2 2nd component from right, model function 3.

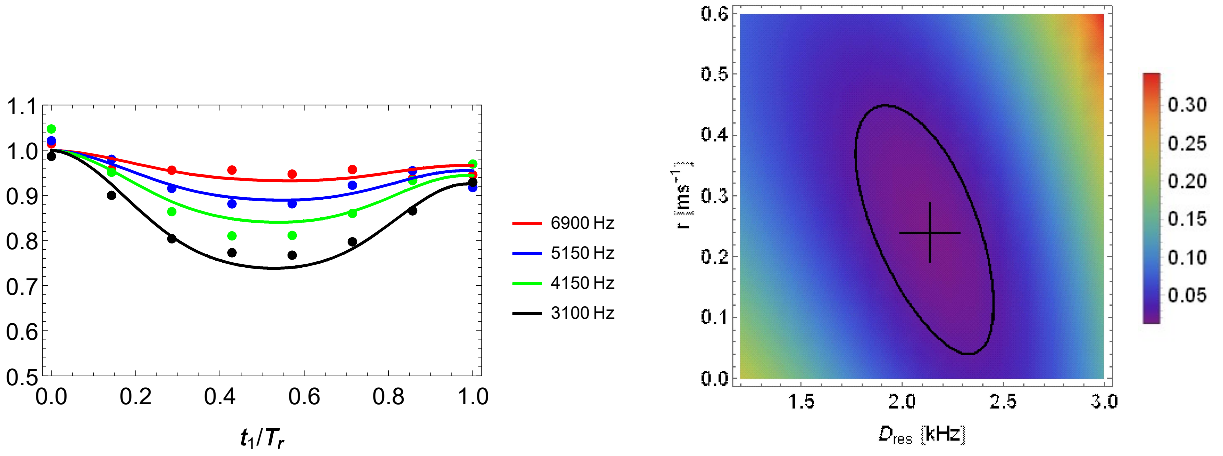

Figure S15. PIZOF-10, 25°C, CH_2 middle component, model function 3.

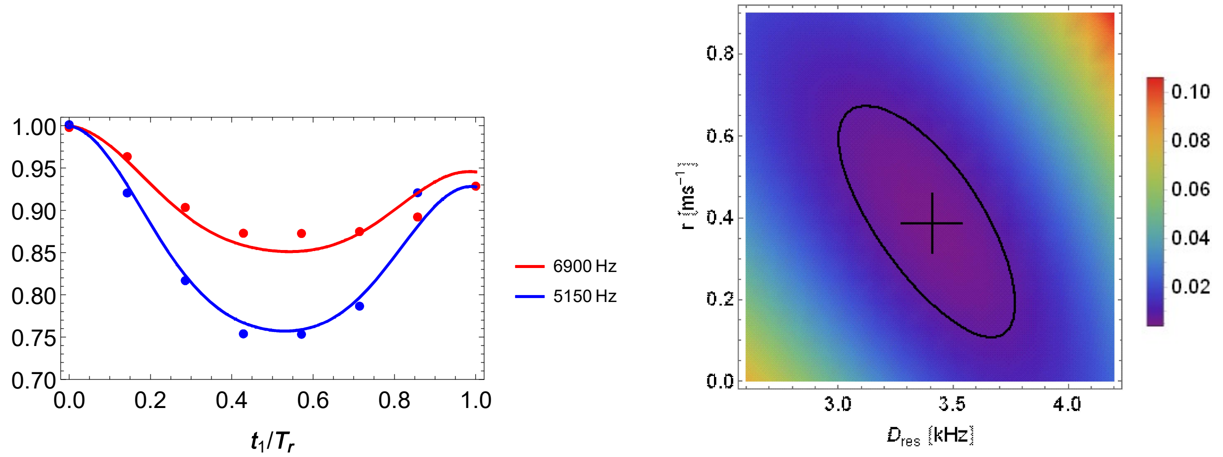

Figure S16. PIZOF-10, 25°C, CH_2 2nd component from right, model function 3.

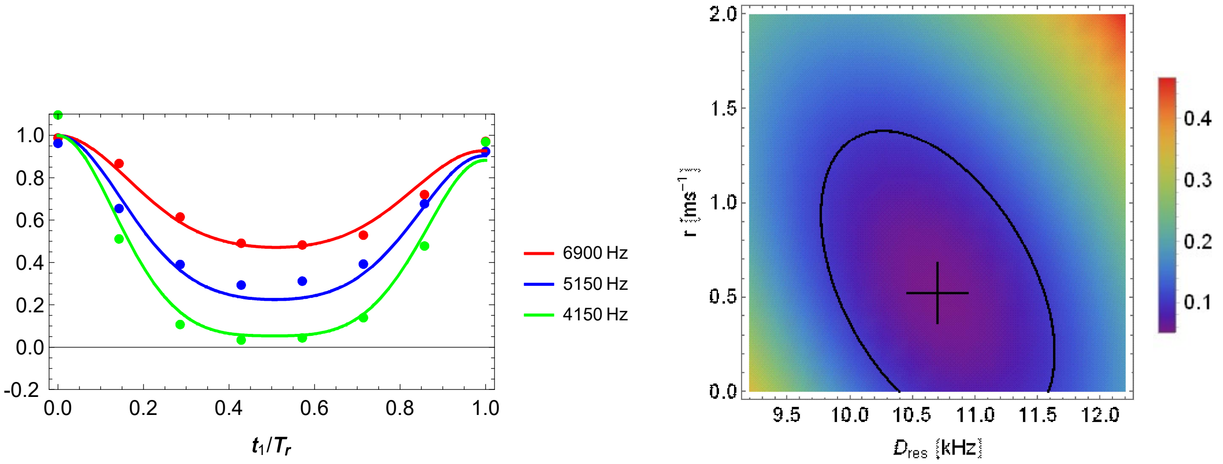

Figure S17. PIZOF-10, 25°C, CH of middle ring, model function 1.

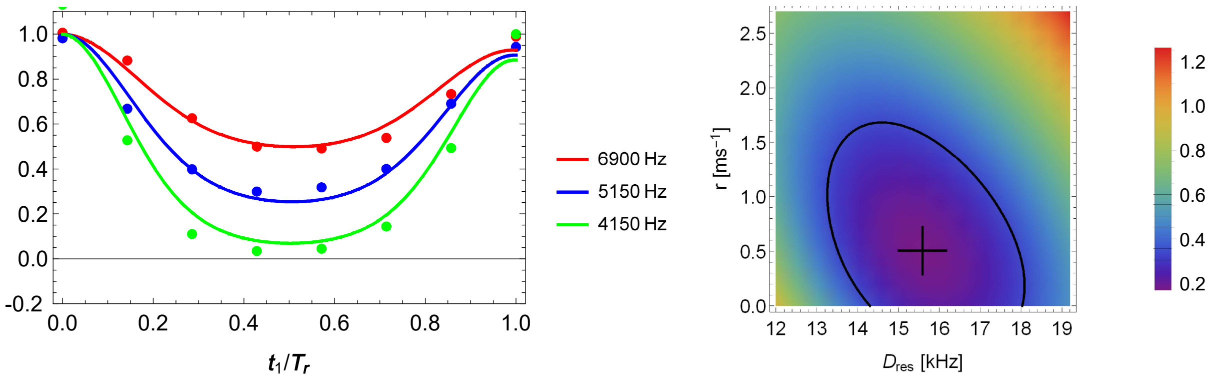

Figure S18. PIZOF-10, 25°C, CH of middle ring, model function 2.

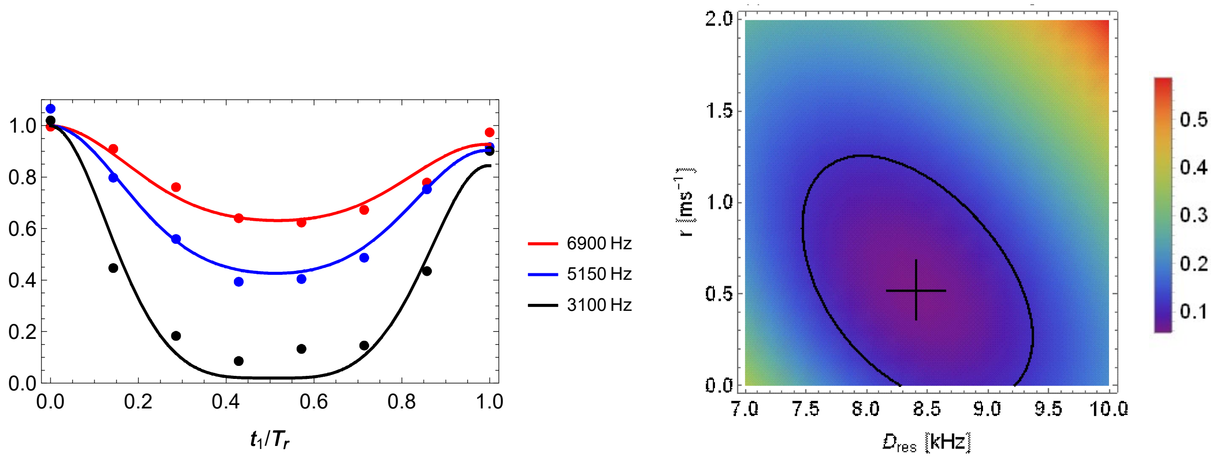

Figure S19. PIZOF-10, 25°C, CH of side ring, model function 1.

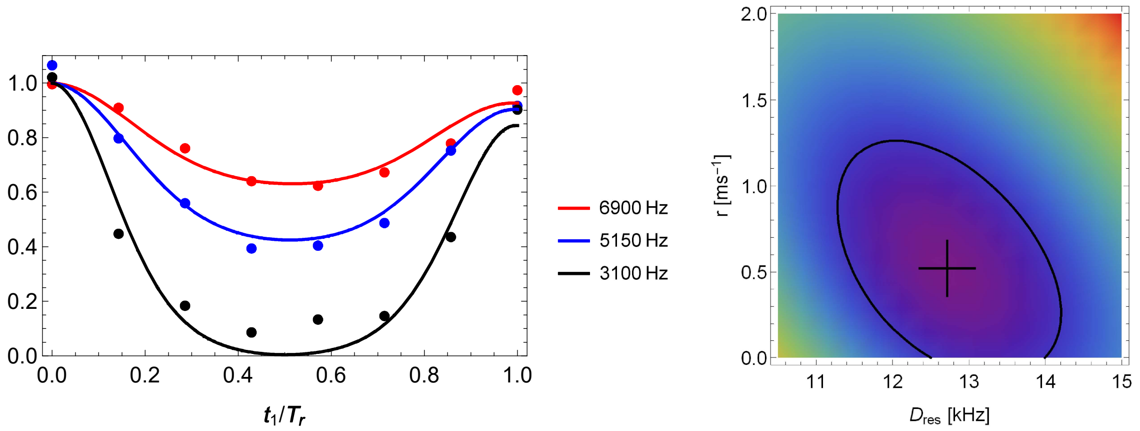

Figure S20. PIZOF-10, 25°C, CH of side ring, model function 2.

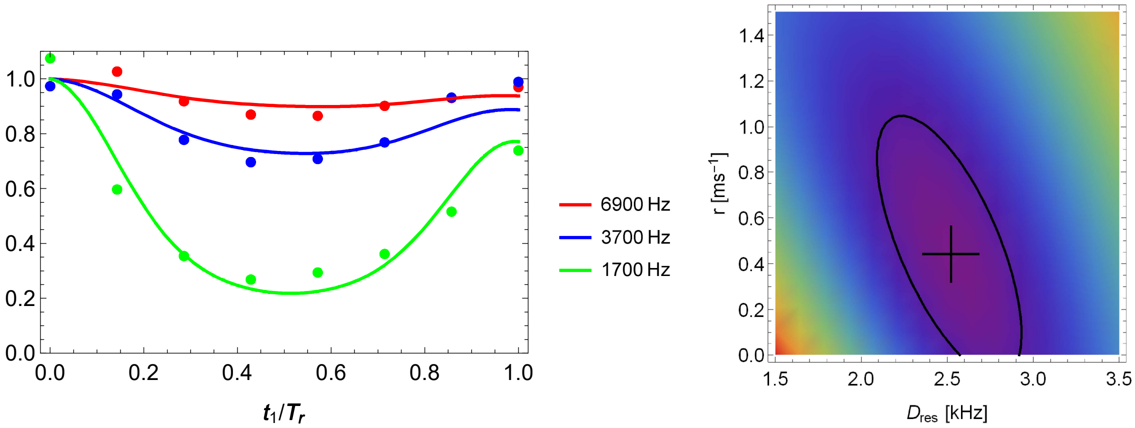

Figure S21. PIZOF-11, -15°C, δ-CH_2 left component, model function 3.

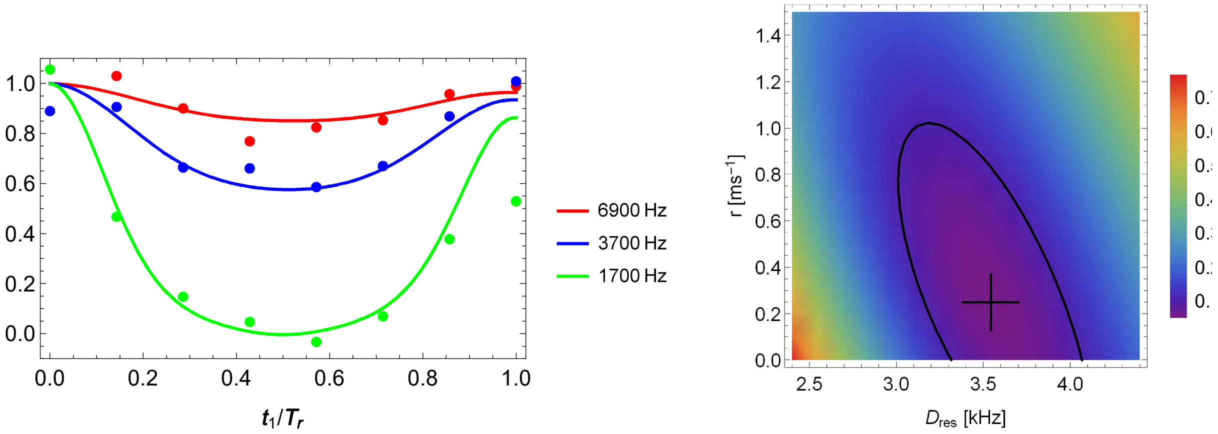

Figure S22. PIZOF-11, -15°C, ε-CH_2, model function 3.

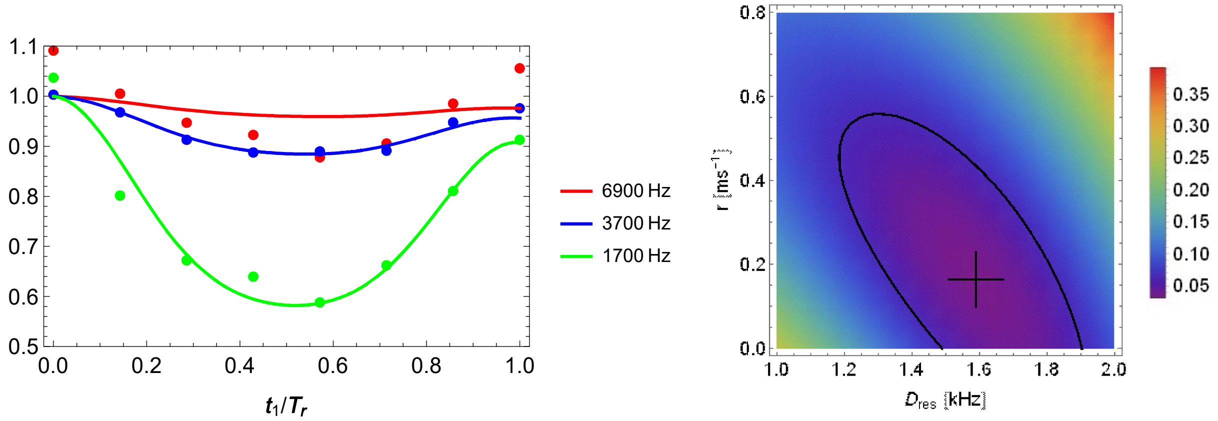

Figure S23. PIZOF-11, 30°C, γ-CH_2, model function 3.

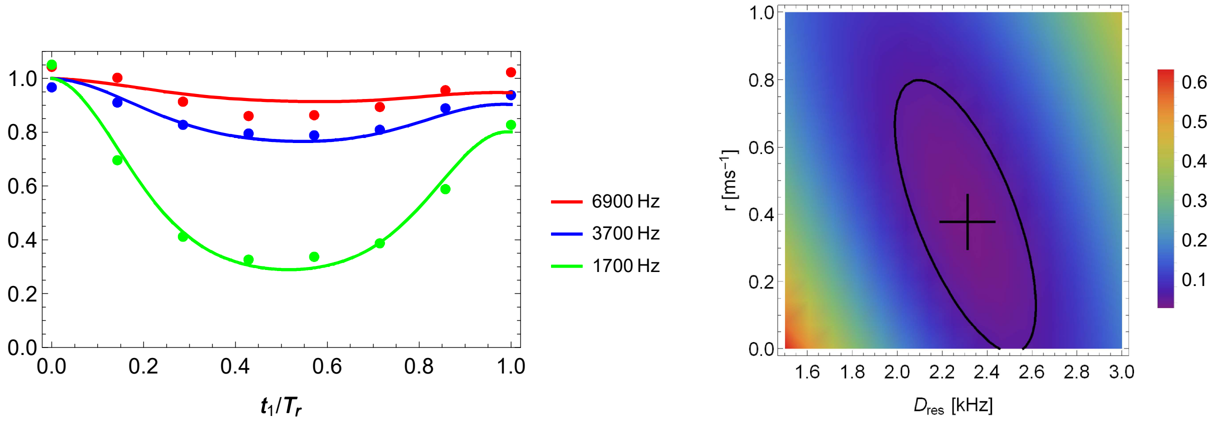

Figure S24. PIZOF-11, 30°C, δ-CH_2, model function 3.

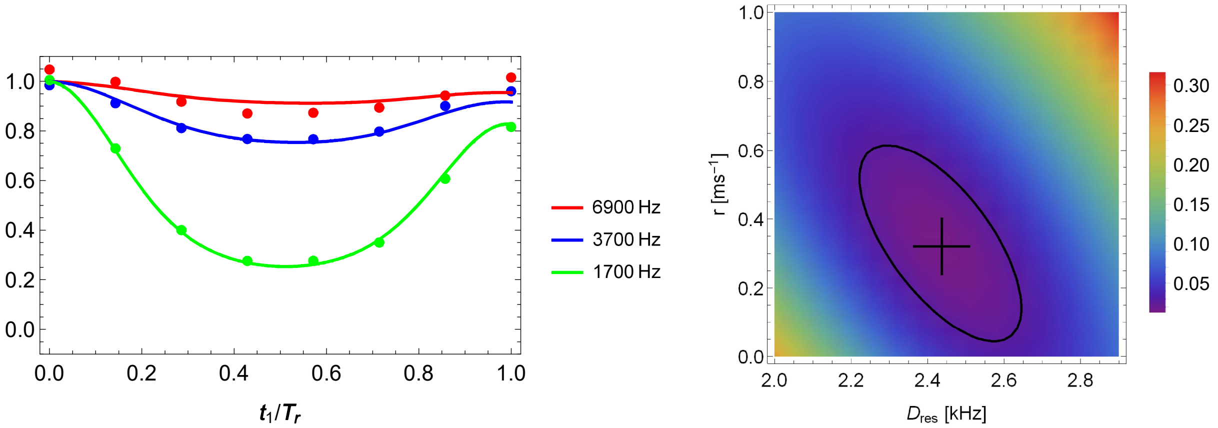

Figure S25. PIZOF-11, 30°C, ε-CH_2, model function 3.

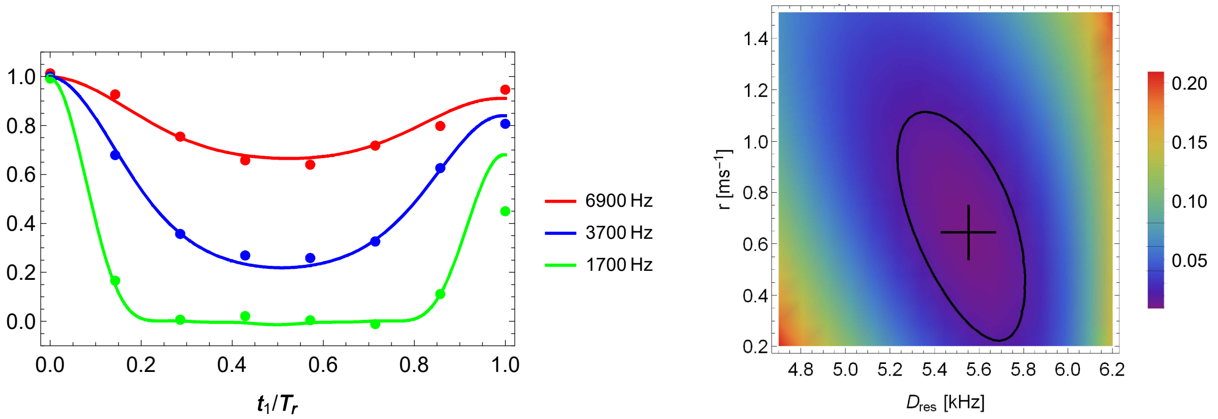

Figure S26. PIZOF-11, 30°C, ω-CH_2, model function 3.

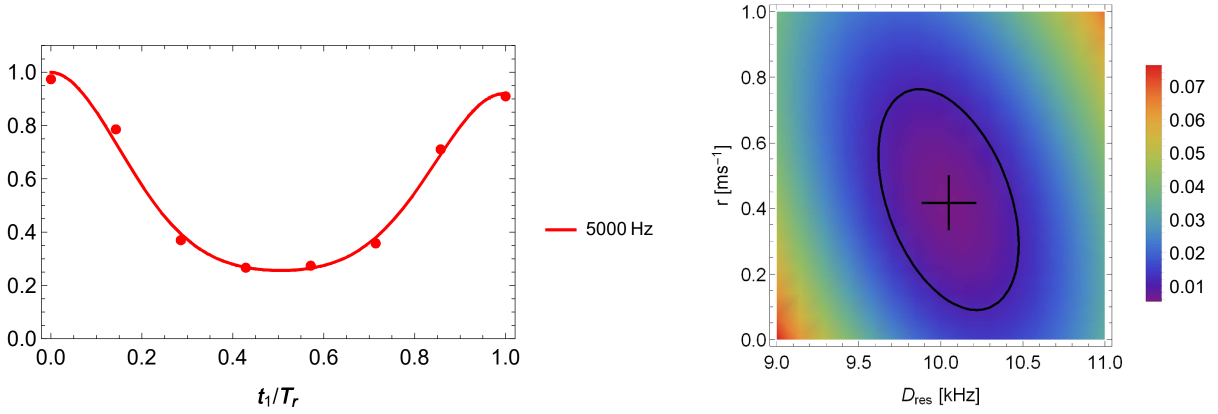

Figure S27. PIZOF-11, 25°C, middle ring, model function 1.

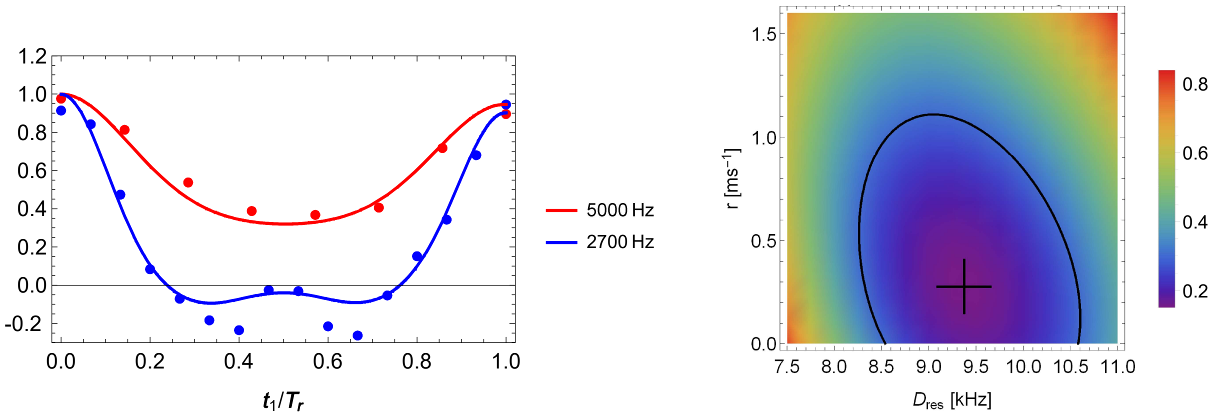

Figure S28. PIZOF-11, 25°C, side ring, model function 1.
